# A Miniaturized Wireless Micropump Enabled by Confined Acoustic Streaming

**DOI:** 10.34133/research.0314

**Published:** 2024-02-26

**Authors:** Rui You, Qian Fan, Zilun Wang, Wenqiang Xing, Yuchuan Wang, Yi Song, Xuexin Duan, Rui You, Yan Wang

**Affiliations:** ^1^ State Key Laboratory of Precision Measuring Technology & Instruments, Tianjin University, Tianjin 300072, China.; ^2^ Tianjin Eye Hospital, Tianjin Key Lab of Ophthalmology and Visual Science, Nankai University Affiliated Eye Hospital, Nankai University Eye Institute, Nankai University, Clinical College of Ophthalmology Tianjin Medical University, Tianjin Eye Institute, Tianjin 300020, China.; ^3^ School of Instrument Science and Opto-Electronics Engineering, Beijing Information Science and Technology University, Beijing 100192, China.; ^4^ Beijing Advanced Innovation Center for Integrated Circuits, Beijing 100084, China.

## Abstract

Miniaturization of health care, biomedical, and chemical systems is highly desirable for developing point-of-care testing (POCT) technologies. In system miniaturization, micropumps represent one of the major bottlenecks due to their undesirable pumping performance at such small sizes. Here, we developed a microelectromechanical system fabricated acoustic micropump based on an ultrahigh-frequency bulk acoustic wave resonator. The concept of an inner-boundary-confined acoustic jet was introduced to facilitate unidirectional flow. Benefitting from the high resonant frequency and confined acoustic streaming, the micropump reaches 32.620 kPa/cm^3^ (pressure/size) and 11.800 ml/min∙cm^3^ (flow rate/size), showing a 2-order-of-magnitude improvement in the energy transduction efficiency compared with the existing acoustic micropumps. As a proof of concept, the micropump was constructed as a wearable and wirelessly powered integrated drug delivery system with a size of only 9×9×9 mm^3^ and a weight of 1.16 g. It was demonstrated for ocular disease treatment through animal experimentation and a human pilot test. With superior pumping performance, miniaturized pump size, ultralow power consumption, and complementary metal–oxide–semiconductor compatibility, we expect it to be readily applied to various POCT applications including clinical diagnosis, prognosis, and drug delivery systems.

## Introduction

In the past few decades, miniaturization of health care, biomedical, and chemical systems in the laboratory has drawn a surge of attention [1–3]. Consequently, novel portable systems, including micro-total analysis systems [4], lab-on-a-chip [5], organ-on-a-chip [6], point-of-care testing (POCT) systems [7], and controlled drug delivery systems (cDDSs) [8,9], have been largely developed. In these portable systems, the flexible manipulation and transportation of small amount of fluid is one of the central requirements [10,11]. Micropumps play a key role in these systems, assisting with the diverse functions, such as molecule detection [12,13], cell culture [14,15], drug screen [16,17], particle manipulations[18,19], etc. It is routine and simple in laboratory conditions; however, it has become one of the major limitations as real portable systems for in-field applications [20–22]. At present, syringe pumps account for the most popular fluid drivers, but are not suitable for applications outside laboratories due to their bulky sizes and inconvenient power supplies [23,24]. Thus, small-size, lightweight, low-power-consumption micropumps are conceptually required for fluid manipulation in these portable systems and have attracted great interest [25–27]. To date, numerous micropumps based on different principles have been developed, such as thermopneumatic [28], piezoelectric [29–41], electromagnetic [42–45], phase-change [46], dielectric elastomer [47], electroosmotic [48], electrohydrodynamic [49], and acoustic [23,50–55] systems. A micropump for a practical portable system should provide sufficient pumping performance without damaging the portability, which, in simple terms, requires the micropump to be small and easy to drive. The existing micropumps, broadly speaking, can be categorized into 2 groups [26,56]: mechanical micropumps with moving elements and nonmechanical micropumps without moving elements. As a representative of mechanical micropumps, the electromagnet micropump has a complex structure, and the rotor requires it to operate in a horizontal and stable position. These factors make its application in portable systems very challenging, especially for wearable devices. As an emerging representative of mechanical micropumps, the piezoelectric micropump has no demands on the working pose but harnesses a high working voltage (>100 V) and check valves to maintain a large flowrate and sufficient differential pressure. As a result, the piezoelectric micropump must work with mains sockets or bulky batteries. The thermopneumatic micropump drives the fluid at a low voltage and can be powered by small lithium batteries but provides small flow rates even at 0 Pa backpressure. The phase-change micropump has a simple structure and generates sufficient differential pressure without external power sources. However, its self-powering method causes this micropump to lack controllability and reusability. Apart from mechanical micropumps, nonmechanical micropumps with no moving elements have been largely developed as well. The elimination of the moving parts usually facilitates the miniaturization and stability of the micropump. However, it brings a higher demand on the power source. Taking electrohydrodynamic, dielectric elastomer, and electroosmotic micropumps as examples, they all need a high voltage (>1 kV) to operate and suffer from complicated power sources with low safety. To date, most mechanical and nonmechanical micropumps are still limited in laboratory usages.

In recent years, acoustic waves induced by the piezoelectric effect have been employed to drive liquids and manipulate particles in a wide range of miniaturized systems (especially microfluidic systems) [57–67]. These piezoelectric chips fabricated via advanced microelectromechanical system techniques [68,69] enable acoustofluidic micropumps (AFMPs) with great portability (simple and small structures, low power consumption, and low working voltage), high biocompatibility, a fast response time, and fair extensibility [23,50–54], which theoretically meets the requirements of a portable system as an ideal micropump. However, the existing AFMPs have unsatisfactory pumping performance due to their limited energy transduction efficiency (Table S2). Recently, gigahertz (GHz) bulk acoustic waves (BAWs) have been demonstrated to induce intense acoustic streaming in a very small area due to the focused acoustic wave and rapid dissipation at the device–liquid interface, which have been applied for different applications, including solution mixing, droplet dispensing, and bioparticle manipulation [59,62,66,67,70,71]. However, for pumping applications, the GHz acoustic streaming tends to be vortical in nature, which prevents the formation of unidirectional flow.

**Fig. 1.**
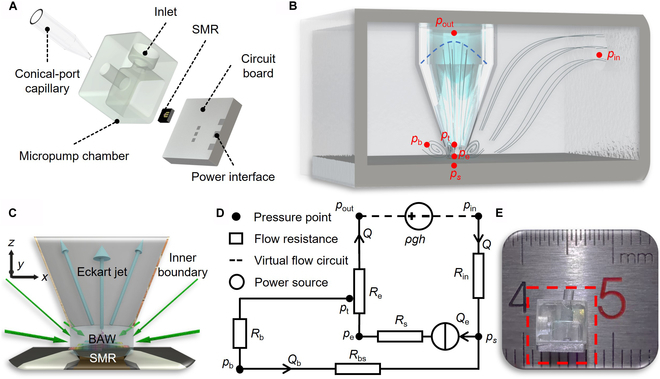
Schematics of AcousJMPs. (A) Assembly schematic of a typical AcousJMP. (B) Diagram of the working flow field of an AcousJMP. (C) Diagram of the ideal acoustic streaming effect for pumping. (D) Diagram of the fluidic circuit of an AcousJMP. (E) Photograph of an AcousJMP with dimensions of 5×5×4 mm^3^.

As mentioned, there is still a lack of suitable micropumps to meet all the requirements for POCT applications. In this article, we developed an acoustic jet micropump (AcousJMP) (Fig. 1A and E) based on GHz acoustic streaming. An inner boundary confinement concept is introduced for the first time to regulate the acoustic streaming, which enables the unidirectional flow. We discussed the design rules of the inner boundary confinement in detail. The suitable isolation of the acoustic jet from the formation of acoustic vortices endows the AcousJMP with superior pumping performance. The minimized pump size and ultralow power consumption largely facilitate the integration of the micropump for POCT applications. As a proof of concept, we constructed an AcousJMP as a wearable and wirelessly powered integrated drug delivery system (wDDS) for ocular disease treatment. The as-developed wDDS was tested in animal experiments as well as in a human pilot test. The results obtained in this work supports the possibility of developing more advanced AFMPs and impels the miniaturization and commercialization of novel personalized health care technologies.

## Results

### Working principle of GHz AcousJMPs

**Fig. 2.**
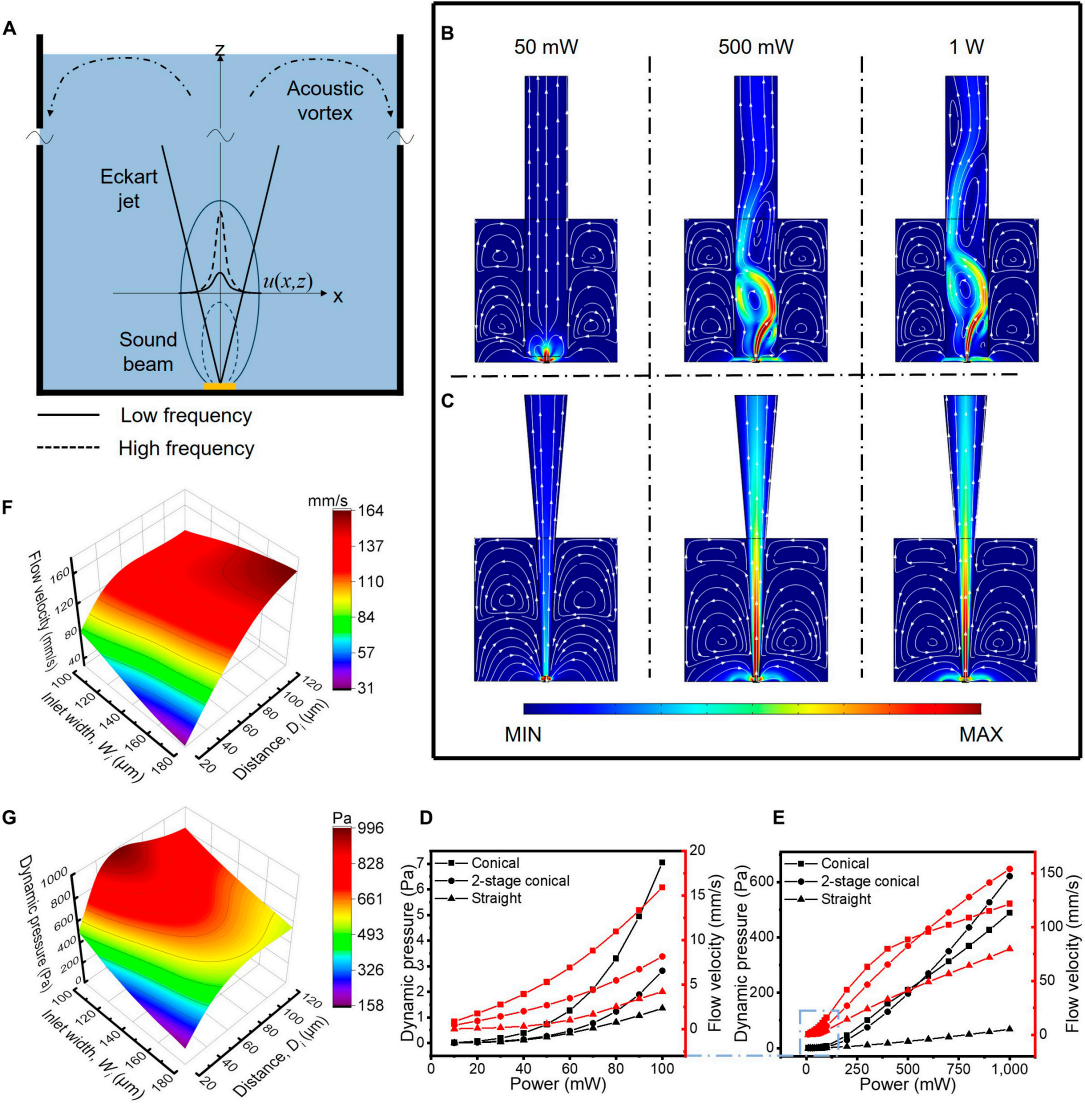
Simulation results of the regulation of the inner boundary situation to adjust the acoustofluidic field. (A) Diagram of the mechanism of high-performance jetting using the acoustic streaming effect. Regulation of different inner boundary situations (B: straight; C: 2-stage conical) at different powers. (D and E) Changes in the dynamic pressure and flow velocity in different inner boundary situations when the power varies. Influence of the bottom width and height on the pumping performance, flow velocity (F), and dynamic pressure (G).

Once a radio frequency (RF) signal is received, the BAW oscillates in thickness mode, transforming the electrical energy into acoustic energy through the inverse piezoelectric effect. Then, the longitudinal acoustic wave immediately propagates into the fluid because the BAW directly contacts the fluid, thus avoiding the energy losses associated with solid–liquid interfaces and dissipation during transmission through solid media. As the acoustic waves propagate in the fluid medium, they induce the fluid to oscillate and cause acoustic pressure, *p*_1_, in the fluid domain, Ω [72]:

$$\nabla^{2}p_{1}+k_{c}^{2}p_{1}=0$$ (1)

where *k_c_* is the complex-valued compressional wavenumber. From *p*_1_, the acoustic velocity, ***v****_1_*, can be obtained:

$$v_{1}=\frac{-i\left(1-i\Gamma \right)}{\omega \rho }\nabla p_{1}$$ (2)

where *ω* is the angular frequency of the acoustic wave, *ρ* is the liquid density, and Γ is the weak bulk damping coefficient. Due to viscous dissipation, relaxation effects, and turbulent fluctuations in the fluid, the first-order fields produce the nonzero time-averaged second-order fields. The second-order acoustic streaming is directly driven by the acoustic body force, ***F****_b_*, which can be obtained from *p*_1_ and ***v***_1_:

$$F_{b}=\frac{\Gamma \omega }{c^{2}}\langle {\tilde{p}}_{1}{\tilde{v}}_{1}\rangle$$ (3)

where *c* is the acoustic speed in the fluid and “~” represents its complex-valued amplitude. Subjected to ***F****_b_*, the fluid above the BAW becomes a momentum flux coaxially emanating from the sound beam, which is called Eckart streaming [73]. As Γ∝*ω*, this acoustic body force increases with *ω*^2^, indicating that the higher the frequency is, the stronger the jet. Referring to [74], the finite edge of the jet is defined as the aggregation of the point at which the velocity is 1% of its axial value. As shown in Fig. 2A, due to the viscous entrainment of the adjacent liquid, the jet edge gradually becomes beyond the acoustic source edge along its transmission direction. Then, the streaming field tends to be vortical in nature, rotating around a point in each longitudinal section. This consumes a large proportion of energy, which is similar for all acoustofluidic systems. Therefore, to reduce the energy dissipated across the internal load of the AFMP and improve the energy utilization coefficient, it is necessary to design a suitable inner boundary situation to regulate the streaming field.

Through analysis of the acoustic pressure (Fig. S2B), the momentum flux (*Q_e_*) within a small space is almost entirely actuated by only ***F****_b_* and immune to other components of the circuit to a marked extent; thus, it is regarded as a constant flow source (Fig. 1C). In addition, the differential height of fluid levels exerts a gravitational pressure, so it can be seen as a virtual pressure source without fluid flow. Therefore, each typical acoustofluidic system can be simplified as a fluidic circuit containing 2 loops (internal and external loops) and 2 power sources (Fig. 1B and D). Referring to Ohm’s law and the linear superposition theorem in digital circuits, the flow rates in the fluidic circuit, *Q* and *Q_b_*, can be derived as:

$$Q=Q_{1}+Q_{2},Q_{b}=Q_{b1}+Q_{b2}$$ (4a)

$$Q_{b1}\left(R_{b}+R_{\mathrm{bs}}\right)=Q_{1}\left(R_{\text{in}}+R_{e}^{\prime }\right)$$ (4b)

$$Q_{1}=Q_{e}-Q_{b1}=Q_{e}\left(R_{b}+R_{\mathrm{bs}}\right)/\left(R_{b}+R_{\mathrm{bs}}+R_{\text{in}}+R_{e}^{\prime }\right)$$ (4c)

$$Q_{2}=-Q_{b2}=-\rho \mathrm{gh}/\left(R_{e}^{\prime }+R_{\text{in}}+R_{b}+R_{\mathrm{bs}}\right)$$ (4d)

where $R_{e}^{\prime }$ is the part of *R_e_* between *p_t_* and *p_out_*, and the subscripts “1” and “2” represent the constant flow source and the virtual pressure source, respectively. In conventional AFMPs, the resistances of the internal loop (*R_b_*, *R_bs_*, etc.) share the same fluid space as those of the external loop ($R_{e}^{\prime }$, *R_in_*, etc.), and the former are much smaller than the latter due to the finite width of the jet. This, from the view of the fluidic circuit, also explains why most of the jet gradually joins the vortices, providing a poor pumping performance. To improve the pumping performance and bring the AFMP into practical utilization, we first introduce the concept of the inner boundary situation in the AFMP, which can be adjusted to isolate the jet from vortices and restrain the formation of vortices, achieving balance of the flow resistance network in the fluidic circuit.

Herein, we combined the 2D finite element method (FEM) with the fluidic circuit model to illustrate the regulation of the inner boundary situation for an Eckart jet without a height difference between fluid levels (*h(t)* = 0). We created a symmetric 2D simulation model where a capillary was inserted along the axis of symmetry (*z*-axis) into the fluid chamber to form the inner boundary situation (Fig. S2A). The BAW is 140 μm wide (smaller than the diameter of the inscribed circle of a 20-kμm^2^ BAW of 148 μm), the fluid chamber is set as 3×3 mm^2^ with 2 open ports, and the capillary has 3 important parameters: the width of the middle part (*W_m_*), the width of the inlet (*W_i_*), and the distance from the BAW (*D_i_*). When the micropump works without a height difference, the pressure can be written as:

$$p=\mathrm{QR}+\rho \mathrm{gh}=Q\left(R_{\text{in}}+R_{s}+R_{e}\right)$$ (5)

which can be reflected by the dynamic pressure at the inlet to a certain extent.

First, the acoustic field was simulated under different powers. As shown in Fig. S2B, 1.596-GHz acoustic waves quickly attenuate along the propagation direction, and their energy concentrates in a very small area and is transformed into fluidic kinetic energy through acoustic streaming effects. Then, the acoustic streaming would induce the flow resistances, forming the fluidic circuit. Similar to an electrical resistance, the flow resistance is positively associated with the flow length and negatively associated with the cross-sectional area of the flow. Referring to these rules, the fluidic circuit can be flexibly tuned. In the following part, 4 types of boundaries are analyzed and compared to demonstrate the suitable boundary situation.

In the first case, a straight capillary (*W_m_* = 900 μm, *W_i_* = 900 μm) is inserted 100 μm from the BAW. The width of the jet in the inlet is smaller than the width of the inlet. This leaves large space for vortices, making *R_bs_* and *R_b_* small. Consequently, *Q_b_* is larger than *Q*. As shown in Fig. 2B, only a small amount of the jet escapes from the vortices and achieves unidirectional flow at 50 mW. When the applied power is increased, the Eckart jet, *Q_e_*, becomes stronger, and the vortices also become stronger. This, in turn, increases the flow resistances, *R_in_*, *R_e_*, *R_bs_*, and *R_b_*. Thus, the flow velocity and the dynamic pressure both increase as the power increases (Fig. 2D and E). In the second case, the inlet is blocked, leaving a slit with a width of 160 μm (Fig. S3A). Obviously, the jet occupies most of the slit, greatly restricting the vortices at a low power (50 mW). This causes *R_bs_* and *R_b_* to become large while also increasing *R_in_*. As the power increases (500 mW, 1 W), the vortices enter the capillary and become larger, where *R_bs_* and *R_b_* decrease. Therefore, the flow field becomes similar to that in the first case. Next, we investigate the shape effect of the inlet. When the inlet is shaped like a cone with a width of 160 μm (Fig. S3B), it allows a large space for *R_in_*. Different from the flow fields at low power in the previous 2 cases, the flow field in this case successfully achieves the separation of the jet and vortices, with large *R_bs_* and *R_b_* and small *R_in_*. As shown in Fig. 2D and E, this conical inlet performs much better than the straight inlet in a wide power range of 10 to 1,000 mW. Note that minor loops appear in the conical capillary when the power is greater than 500 mW. This results in slowdown of the flow velocity growth. Hence, we then adopt a 2-stage conical capillary, in which the middle part is narrowed to 400 μm based on the conical capillary. This situation, called a 2-stage conical capillary, provides a larger $R_{e}^{\prime }$ than the conical capillary, thus impeding the formation of minor loops (Fig. 2C). Compared with the conical capillary, the 2-stage conical capillary provides a poorer performance at a low power but a better performance at a high power (Fig. 2D and E). Based on these analyses, making the lateral boundaries close to the edge of the jet can enlarge the flow resistances of the internal loop and restrain the vortices (especially at a high applied power), achieving the desirable pumping performance.

We then studied the influence of *D_i_* on the flow field and the resistance network. As shown in Fig. S4A, the straight capillary is placed 400 μm from the BAW. At a low power (10 mW), the small *R_in_*, *R_bs_*, and *R_b_* lead to unconstrained vortices, which is similar to the cases without inner boundary confinement. As the power increases, the jet becomes stronger, limiting the vortices with the assistance of inner boundaries. This limitation is not provided by the inner boundaries alone. In other words, it is fragile. For example, when the micropump transmits liquid between 2 chambers with height difference (*h* ≠ 0), the small resistances ($R_{e}^{\prime }$, *R_in_*, *R_bs_*, and *R_b_*) would allow a large counterflow against the jet flow, which means a small *Q* but a large *Q_b_*. This implies that this boundary situation is only suitable for those systems with small load resistances and without a height difference (Fig. S5). However, we note that this trend is impeded in the conical inner boundary situation (Fig. S4B) owing to the exclusion of vortices from the capillary. In summary, a large *D_i_* can increase the flow velocity but reduce the differential pressure (Fig. 2D and E and Fig. S5), which provides better pumping performance in a system with a low backpressure.

Next, we investigated the parameters of the inlet in the 2-stage conical inner boundary situation in detail due to its consistency and high performance over a wide power range (Fig. 2E). Based on the above simulation results, *W_i_* is increased from 100 to 180 μm in a 20-μm step, and *D_i_* is increased from 20 to 120 μm in a 20-μm step. In these situations, the vortices are almost pulled out of the capillary. These situations are simulated at 1 W. As plotted in Fig. 2F, the flow velocity grows as *D_i_* increases, which reduces *R_in_* and increases *R_bs_* and *R_b_* because these flow resistances share the same space. When *D_i_* is small (20 to 60 μm), a wider inlet leads to a smaller flow velocity. While when *D_i_* is large (100 and 120 μm), a wider inlet leads to a larger flow velocity. This is because the former setup fits better to the Eckart jet; thus, the former allows the fluid to smoothly flow. In contrast, the results in Fig. 2G illustrate that the narrow inlet can capture the fast part of the jet, resulting in a larger dynamic pressure than wide inlets. The dynamic pressure drops when *D_i_* is so small that *R_in_* becomes high, which should be considered in micropump design and fabrication.

**Fig. 3.**
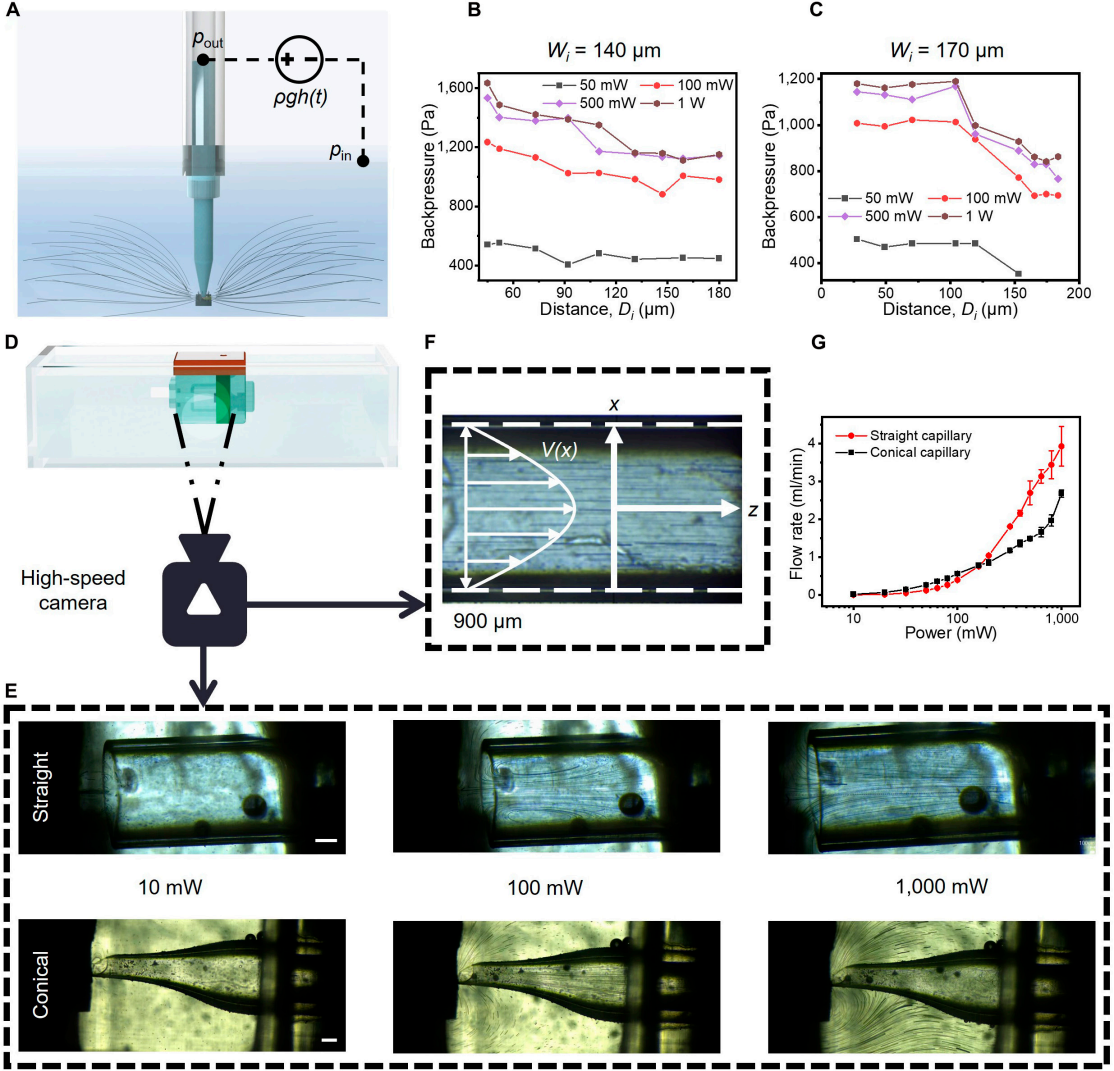
Pumping performances of AcousJMPs with different inner boundary situations. (A) Diagram of the experimental setup for regulation of the inner boundary situation to adjust the fluidic circuit and the backpressure. Backpressures provided by conical capillaries with different inlet widths (B, 140 μm; C, 170 μm) and heights. (D) Diagram of the experimental setup for regulation of the inner boundary situations to adjust the acoustofluidic field. (E) Acoustofluidic field under different inner boundary situations (top, straight; bottom, conical). Scale bar: 200 μm. (G) Flow rates of AcousJMPs with different capillaries, which are estimated according to the parabolic distribution of the flow velocity in the outlet of the capillary (F).

Finally, we conducted 3D simulation and directly set the (2-stage) conical capillary above a BAW to realize the inner boundary situation in an open system to test its performance of the differential liquid pressure, which is the major issue in existing AFMPs. Here, *D_i_* could be tuned by a precision displacement platform (Fig. 3A and Fig. S6). The pressure source here is calculated with reference to [52], which is demonstrated in the Supplementary Text and Fig. S7 in detail. For this experiment, there are 2 main differences from the 2D simulation model: (a) *h* is not always zero; (b) $R_{e}^{\prime }$ increases with increasing *h*. When the diameter of the conical inlet (referring to *W_i_*) is 140 μm, *D_i_* is tuned from 45 to 180 μm. As a result, the maximum backpressure decreases with increasing distance (Fig. 3B). The larger the power is, the steeper the drop in the backpressure. In another case where *W_i_* is 170 μm, *D_i_* is tuned from 27 to 180 μm (Fig. 3C). Because of the wider inlet, this situation generates a lower differential pressure than the former. These results are exactly what we speculate according to the simulation results and guide the following design and fabrication of AcousJMPs.

### Design, fabrication, and characterization of AcousJMPs

Based on these analyses and results, a typical AcousJMP is designed to consist of 5 parts: a GHz BAW resonator (1.5 to 1.6 GHz, Fig. S1) as the actuator to induce strong flow, a conical-port capillary with a microscale inlet for providing the boundary situation to regulate the flow, a micropump chamber as support, a power interface to receive the drive signal, and a circuit board for BAW loading and future function extension. Movie S1 shows the assembly process of a wireless AcousJMP in detail. Considering convenient integration with the antennas, we designed and fabricated 2 AcousJMPs (9×9×8 mm^3^) (the AcousJMP with a straight capillary: *D_i_* = 400 μm, *W_i_* = 900 μm; the AcousJMP with a conical capillary: *D_i_* = 200 μm, *W_i_* = 160 μm). The pump body was made of the clear resin, creating viewing windows on 2 sides to observe the internal flow field. The capillary was made of clear glass to showcase the inner flow field. Prior to each experiment, the AcousJMP was fixed and filled with ultrapure (UP) water. During this experiment, referring to the FEM model, the AcousJMP was immersed in a glass sink fully filled with water containing seeding spheres of 10 μm diameter to aid flow visualization (Fig. 3D). A high-speed camera was positioned to record the acoustic streaming field.

As shown in Fig. 3E, when the AcousJMP with a straight capillary works at a very low power (10 mW), the Eckart jet is almost transformed into vortices with little output flow, which is exactly predicted by the fluidic circuit and the FEM model (Figs. S4 and S5). Next, the flow velocity at the outlet of the capillary is assumed to have a parabolical distribution (Fig. 3F) due to the small Reynolds number. Then, the flow rate can be estimated. As shown in Fig. 3E, because the wide inlet allows *R_b_*, *R_bs_*, and *R_e_* to share the space, the strong Eckart streaming and the capillary enlarge *R_bs_* and *R_b_* as the power increases. This results in a considerable flow rate of 3.926 ml/min at 1 W (Fig. 3G). Experimentally, the maximum backpressure of the AcousJMP with a straight capillary could be assessed by connecting a silicon tube to its outlet (Fig. S8A). After 10 min operation at 1 W, the height difference, *h*, stabilizes at ~9 mm, and the maximum backpressure is estimated to be 97 Pa (Fig. S8B). This pressure only meets the requirement of the low backpressure cases. To further improve the back pressure, the AcousJMP with a conical capillary was designed. As shown in Fig. 3E, by pulling vortices out of the capillary, this 2-stage conical inlet provides small *R_in_*, and large *R_bs_*, *R_b_*, and *R_e_*, generating a larger flow rate than the straight inlet at a low power and a smaller flow rate at a high power (Fig. 3G). Consistent with the dynamic pressures in the simulation results, the AcousJMP with a conical capillary has an excellent pressure performance (3.16 kPa at 1 W, Fig. S8B), overcoming the biggest issues of existing AFMPs. Without considering further extension, we fabricated AcousJMP I (5×5×4 mm^3^, *W_i_* = 200 μm, *D_i_* = 40 μm) with high-pressure performance, low power consumption, and easy integration with portable systems. As shown in Fig. S9A and B, the pressure generated by AcousJMP I linearly increases from 0.313 kPa at 100 mW to 3.262 kPa at 640 mW. The flow rate gradually slows as the differential height rises (Fig. S9C). As the power increases, the maximum backpressure becomes increasingly larger (Fig. S9B). These results highlight the great balance between the portability and pumping performance of the novel AcousJMP.

### AcousJMP-based ocular drug delivery system

**Fig. 4.**
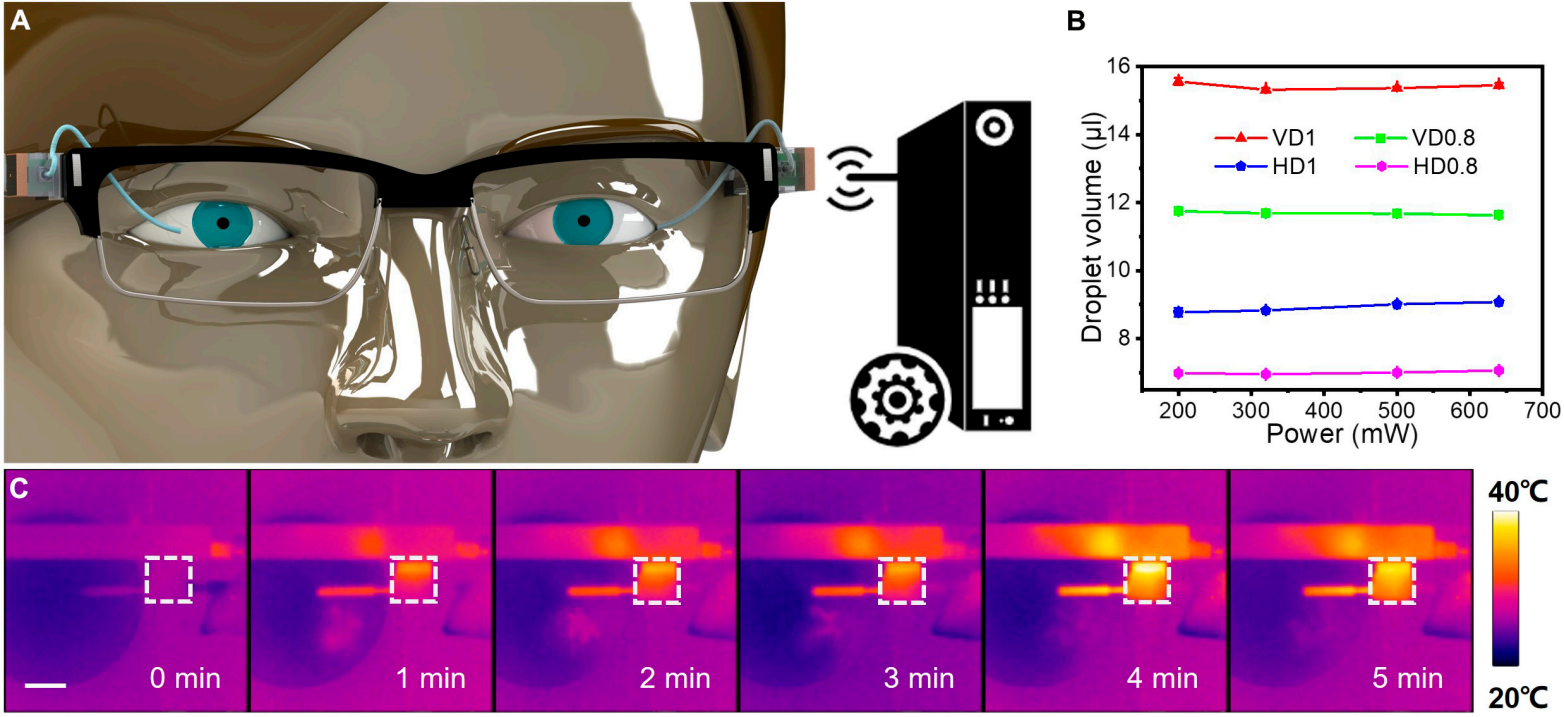
AcousJMP-based wireless oDDS. (A) Wireless AcousJMP-based oDDS for the administration of eye drops. (B) Droplet stability at different catheter interfaces. (C) IR images of the wireless AcousJMP pumping liquid out of the catheter. Scale bar: 1 cm.

To demonstrate the practical application of the micropump, we developed an AcousJMP as a personal wearable medical device for treatment of eye diseases. For most eye diseases, such as glaucoma, dry eye disease (DED), conjunctivitis, and corneal injury, topical administration is the most suitable method used in therapies and has the advantages of noninvasiveness, simplicity, and few side effects. Among all topical administration methods, eye drops are the most convenient, accounting for 90% of commercial ophthalmic dosage forms [75]. However, due to the unique physiology and anatomy of the eye [76,77], eye drops have some shortcomings, including low bioavailability and permeability, short residence time on the ocular surface, frequent usage, and heavy dependence on patient compliance, resulting in low delivery efficiency. Thus, developing advanced eye drop delivery devices to enhance the efficiency of existing eye drops for target patients is necessary. However, the existing micropumps cannot meet the requirements of wearable DDSs (Tables S1 and S2). The AcousJMP is completely qualified for these requirements owing to its advantages of a small size, a light weight, superior pumping performance, a high energy efficiency, and low required voltages and powers. We intended to integrate AcousJMPs with a catheter to deliver eye drops onto the ocular surface without contact, assisting patients in completing topical administration (Fig. 4A).

**Fig. 5.**
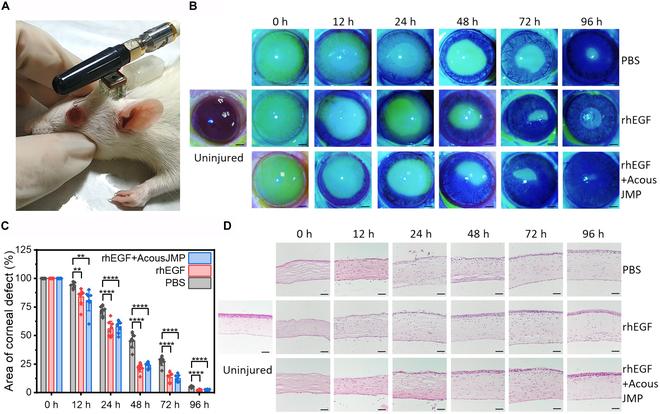
Application of the AcousJMP in a corneal epithelium injury model. (A) Photograph of the therapeutic process of an AcousJMP driving a drug onto the rat’s ocular surface. (B) Slit-lamp micrographs of fluorescein-stained corneas in the 3 groups at the indicated time points after injury. Scale bar: 1 mm. (C) Bar graph showing normalized quantification of the corneal epithelial defect area after drug usage in the 3 groups. Data are shown as the mean ± standard deviation and are representative of 3 independent experimental groups, *n* = 8. *P* values were determined by the 2-sided *t* test. **P < 0.01, ****P < 0.0001. (D) Histopathological changes in rat corneas stained with H&E after injury in 4 days. Scale bar: 50 μm.

The catheter equipped with the AcousJMP-based ocular drug delivery system (oDDS) was fabricated with a small size (9×9×9 mm^3^) and a light weight (1.16 g) and could be wirelessly powered via an antenna (Fig. 5A). Before utilization in eye disease therapies, the precision and thermal safety were evaluated. According to the droplet flow discretization concept, the precision can be stably tuned by the outlet situation. As shown in Fig. 4G, catheters with different outer diameters (1 and 0.8 mm) were connected to a wired AcousJMP and then placed vertically and horizontally above a sink filled with UP water respectively (labeled VD1, VD0.8, HD1, and HD0.8). Through a high-speed camera, the volumes of droplets could be estimated, as shown in Fig. S10A. With increasing power from 200 to 640 mW, the droplet maintains the same volume (Fig. 4B). Obviously, the delivery precision is dependent on the outlet situation but independent of the power. With increasing power, the droplet generation time accelerates (Fig. S10B), providing a maximum of 1.34 ml/min at 640 mW in VD1 (Fig. S10C).

Later, the wireless AcousJMP-based oDDS was activated for 5 min to evaluate its safety through a thermal imager (DT-980, CEM, China) at room temperature, which was much longer than its designed working time in therapies (<1 min per time). As the infrared radiation (IR) images in Fig. 4C show, when the wireless AcousJMP pumps UP water for 1 min, the maximum temperature appears at the antenna, increasing from 27.1 °C to 34.8 °C. The maximum temperature of the chamber increases from 26.9 °C to 31.7 °C. When the wireless AcousJMP works for 5 min, the maximum temperatures in the antenna and the chamber are 38.3 °C and 36.2 °C, respectively. Furthermore, we replaced the UP water with polyvinyl alcohol eye drops to conduct a thermal safety test. These eye drops absorb more acoustic energy and transform it to thermal energy (Fig. S11A), resulting in a maximum temperature increasing from 28 °C to 29.6 °C at 1 min and to 40.4 °C at 5 min (Fig. S11B). These results suggest that the wireless AcousJMP has a desirable thermal safety to meet the requirements of wearable DDSs. By setting a suitable interval between 2 working times, the controlled drug delivery can be achieved for a long time.

### Application of wireless AcousJMPs in the treatment of corneal epithelium injury

To test the systemic biocompatibility and determine the drug delivery efficiency of this wireless AcousJMP-based oDDS, a corneal epithelium injury model was successfully constructed in adult female Sprague–Dawley (SD) rats (Materials and Methods). The proliferation and regeneration of new corneal epithelium occurred for the SD rats, which were divided into 3 groups: one group treated with 1× phosphate-buffered saline (PBS), one group treated with recombinant human epidermal growth factor (rhEGF) by hand, and one group treated with rhEGF by a wireless AcousJMP-based oDDS with the catheter interface (Fig. 5A and Movie S2). As shown in Fig. 5B, the photographs from slit-lamp microscopy demonstrate the process of corneal epithelium dynamic repair after mechanically invasive surgery at different time points: 0, 12, 24, 48, 72, and 96 h. Over time, the corneal epithelium defect areas all recover in the 3 groups. The rhEGF group obviously shows a slight faster recovery than the PBS group. We also noted that the rhEGF+AcousJMP group shows a similar result as the rhEGF group. Next, the whole samples in the 3 groups were all statistically investigated, as shown in Fig. 5C (the fluorescein-stained area [shown in Fig. 5B] at 0 h was set as 100%). The epithelial regeneration in eyes treated with rhEGF is greater than that in the PBS group at 12 to 96 h, demonstrating the efficiency of rhEGF. The rhEGF+AcousJMP group shows similar therapeutic efficiency to the rhEGF group. To further determine the extent of wound repair, the changes in corneal epithelium thickness were measured to assess the depth of the epithelial defect. Through hematoxylin and eosin (H&E) staining, Fig. 5D shows the changes in the histologic appearance of the healing cornea in the 3 groups over time. Epithelial regeneration in eyes treated with rhEGF/rhEGF+AcousJMP is faster than that in eyes treated with 1×PBS at 12 to 96 h. In addition, the AcousJMP has no negative effect during the whole therapy, proving its systemic biocompatibility and the potential of smart oDDSs.

### Human pilot test of the wireless AcousJMP-based oDDS

**Fig. 6.**
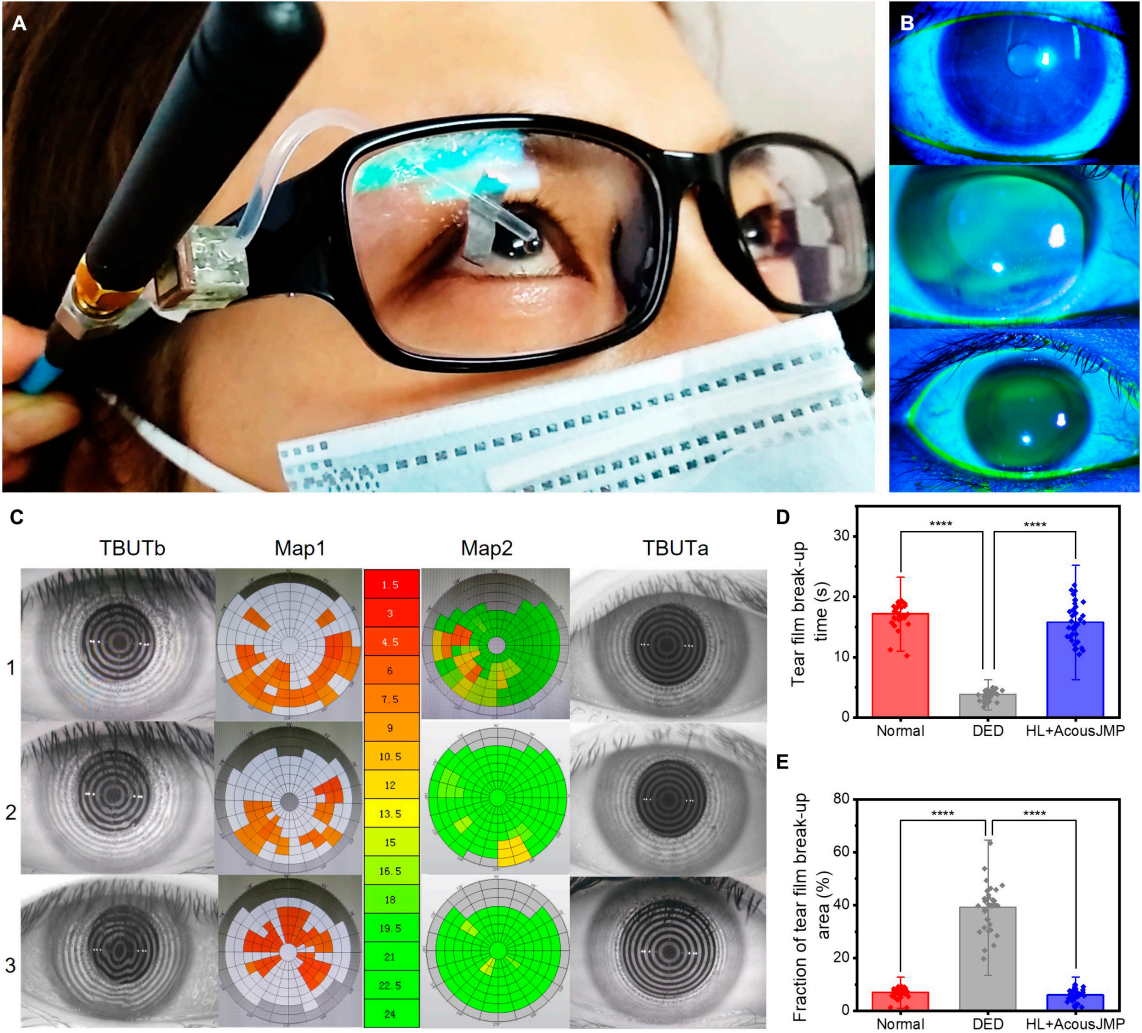
Medical application of AcousJMPs in DED therapy. (A) Photograph of the wireless wearable AcousJMP-based DDS applied in DED therapy. (B) Slit-lamp micrographs showing the progress of corneal epithelium repair after treatment of HL+AcousJMP. (C) Photographs of the spatial mapping of the TBUT in 3 DED patients (Patient 1, Patient 2, and Patient 3). Different colors in the representative circular heatmaps indicate different TBUTs. TBUTb, Map1: before HL+AcousJMP usage; TBUTa, Map2: after HL+AcousJMP usage. (D and E) Bar graphs of the average TBUTs and the fraction areas of the tear film in normal and DED patients before and after HL+AcousJMP. *n* = 12 in the normal, DED, and HL+AcousJMP groups. Data are shown as the mean ± standard deviation of the TBUT and area from the 3 experimental groups. *P* values were determined by 2-sided *t* test. ****P < 0.0001.

Following the application in the therapy for corneal epithelium injury, we developed a wireless AcousJMP-based oDDS for therapy of DED, a major multifactorial and anterior-segmental disease affecting approximately 5% to 50% of individuals in various populations [78,79]. In recent years, the prevalence and burden of DED have grown at a rapid pace, creating an increased need for better therapies to relieve symptoms, e.g., tear replacement, humidification, improved nutrition, and anti-inflammatory ocular agents [80]. Currently, the most common clinical treatment is the topical administration of artificial tear eye drops. However, this treatment is frequent and lengthy; thus, DED patients have difficulty maintaining it in the long term, especially older people, mental health patients, and others with special diseases. To improve the medical service and efficiency for these patients, we first implemented the usage of the wireless AcousJMP-based oDDS in therapy for DED (6 DED patients [NCT04667819]). Figure 6A shows the practical usage of the AcousJMP-based oDDS for a DED patient (Movie S3): through raising of the head to make the antenna close enough to the wireless AcousJMP to receive RF energy, the oDDS delivers eye drops onto the patient’s ocular surface, and once the patients moves away from the transmitting antenna, the received energy decreases and cannot drive the eye drops out of the catheter, showing the quick response time of this oDDS. Figure 6B depicts the process of corneal epithelium repair after treatment with the drug delivered by the AcousJMP-based oDDS through slit-lamp micrographs of human subjects. The corneal epithelium erosion (Fig. 6B, middle) obviously changes after using sodium hyaluronate (HL). To investigate the stability of the tear film, a clinical corneal topography instrument was employed here to monitor its stability and measure the tear film break-up time (TBUT). According to the enrollment criteria and treatment protocol (Materials and Methods), Fig. 6C shows the ring pattern changes on the ocular surface of DED patients (Patient 1, Patient 2, and Patient 3) before (TBUTb, Map1) and after (TBUTa, Map2) treatment with HL+AcousJMP. The ring patterns obviously recover after the usage of HL (TBUTa and Map2) and remain stable for extended periods (10 s). In contrast, the rings generated in the DED group become irregular and unstable more rapidly (TBUTb and Map1). Figure 6D shows the average TBUTs of the 3 groups: normal (17.13 s), DED (3.78 s), and HL+AcousJMP (15.74 s). The statistical quantitative spatial mapping of the TBUT (Fig. 6E) reveals the more rapid break-up of the tear film over a larger surface area in DED patients and further confirms the efficacy of the AcousJMP in the therapy. The results of the normal and HL+AcousJMP groups are not significantly different, and are both significantly different from the results of the DED group. The AcousJMP-based oDDS can be concluded to have potential in DED therapy and to improve medical services for patients with difficulties.

## Discussion

Micropumps are essential for the widespread usage of various POCT applications. As revealed in Table S1, the existing micropumps display unmet pumping performance (pressure and flow rate) per unit volume. The as-developed AcousJMP has high volume efficiencies of 32.620 kPa/cm^3^ and 11.800 ml/min∙cm^3^, both exceeding the reported values (18.431 kPa/cm^3^ in [46] and 11.349 ml/min∙cm^3^ in [39]). Inheriting the advantages of a compact structure, a simple fabrication procedure, and low required voltage and power, the AcousJMP achieves an output pressure in the kPa range, overcoming the biggest issues of current acoustic pumps (Table S2). The high pumping performance and low power consumption together lead to an advanced energy efficiency (*η* = *pQ*/*P_e_*, *P_e_*: the electrical input power [52]), with *η* = 1.84×10^−5^ at *Q* = 0.47 ml/min and *p* = 1.50 kPa when *P_e_* is 640 mW, which is a 2-order-of-magnitude improvement in the efficiency reported for existing acoustic micropumps (*η* = 1.94×10^−7^ at *P_e_* = 2.21 W) [52].

Benefitting from the direct contact with the liquid and the high working frequency, the BAW only needs a very small space to accelerate the fluid, endowing the AcousJMP with great design freedom and universality. Here, we introduced the fluidic circuit model and the 2D FEM model to illustrate the importance of inner boundaries in regulating the fluid field and improving the pumping performance. Simulations of 4 types of capillaries inserted into the micropump chambers suggest the best inner boundary conditions that can isolate the acoustic jet and impede the vortices, achieving the separation of the flow resistances of the internal and external loops. Besides, the distance between the inner boundary and the acoustic source and the regulation by different types of boundaries are thoroughly analyzed. By referring to these, the AcousJMP can be adjusted for various portable applications. As an example, an AcousJMP with a size of only 9×9×9 mm^3^ and a weight of 1.16 g was successfully fabricated to form a wearable oDDS. The oDDS was tested for ocular disease treatment through animal experimentation as well as in a human pilot test, which demonstrated the practical application of the AcousJMP.

In a word, we, in this paper, introduce the concept of inner boundaries into acoustic streaming to fully explore the potential of GHz acoustic streaming as a portable micropump. In future studies, dedicated control units, signal receiving and transmitting units, and sensors could all be integrated into a compact system to develop a “smart pump”.

## Materials and Methods

### Fabrication of a solid-mounted film BAW resonator

Solid-mounted film BAW resonators (SMRs) were fabricated using complementary metal–oxide–semiconductor-compatible technology, and the manufacturing process was reported in previous publications in detail [81]. In brief, a 4-inch silicon wafer was first chosen to be the substrate and cleaned. A Bragg reflector layer was manufactured on it by alternatively depositing aluminum nitride (AlN) and silicon dioxide (SiO_2_). Then, molybdenum (Mo), AlN, and gold (Au) were patterned layer by layer to form a sandwich structure of the resonance area. The schematic fabrication processes and the SEM photograph of an SMR are shown in Fig. S1.

### Fabrication and assembly of a GHz AcousJMP

An SMR (which had been cut into cells of 1×1 mm^2^ using a dicing saw) was fixed on the front of a printed circuit board (PCB) with red glue (under 150 °C) and connected with the circuit through 3 gold wires (1 for the top electrode and 2 for the bottom electrodes), which were sealed with AB glue for protection. A high borosilicate capillary (1 mm outer diameter, YiTai, China) was stretched to be tapered at its fusion point by using a laser-based micropipette puller (P-2000, Sutter Instrument, Novato, CA). Under a high-resolution camera, the conical port was cut to have an end with a diameter of 100 to 200 μm by an ultraviolet (UV) laser cutting machine (CT-UV-5, Beijing Chutianjiguang, China). A micropump body was fabricated using a 3D printer (Form 3, Formlabs Inc., USA) out of photopolymer clear resin (FLGPCL04, Formlabs Inc., USA) as shown in Fig. 1A and D. The walls were smeared with thin layers of photopolymer clear resin on their inner and outer surfaces. Each of the surfaces was covered with a slide and exposed to UV light for 10 min. Then, the side walls would be lucid after moving the slides away, which enables the clear observation in subsequent experiments. The micropump chamber has an outlet (with a diameter of 1.1 mm) on its top, allowing the insertion of the capillary. With the assistance of 3 displacement machines, the micropump chamber and the capillary were placed in correct positions where the axis of the capillary was perpendicular to the SMR surface and above the resonance area with a safe distance. Then, with UV glue (332, Ausbond, USA), the micropump chamber was fixed on the PCB (a thin layer of photopolymer clear resin was cured on the surface of the PCB before this) and the capillary was immobilized on the micropump chamber. The connecting areas were sealed with photopolymer clear resin. Later, for a wireless micropump, an antenna (GPS+BD ceramic antenna, 9×9×1 mm^3^, 1,561/1,575.42 MHz, Boan Tong Xun, China) was placed on the side of the PCB and welded with the circuit by soldering tin, and for a wired micropump, a standard small A type (SMA) adaptor was placed on the side of the PCB and welded with the circuit. Finally, a bottom (if needed) was fixed on the back of the PCB with photopolymer clear resin to form a wireless/wired micropump. The movie S1 shows the assembly process of a wireless AcousJMP.

### Simulations of AcousJMPs

The simulations of AcousJMPs were performed through a commercial finite element analysis software, COMSOL Multiphysics (Burlington, MA, USA). Here, 2D models were built for simplifying calculation (Fig. S2A). For an AcousJMP simulation, a Solid Mechanics module and an Electrostatic module were coupled through a Piezoelectric Effect multiphysics module, achieving the transform from electric energy to mechanical energy. Through an Acoustic-Structure Boundary multiphysics module, the Solid Mechanics module and the Pressure Acoustics module were coupled later, transforming the mechanical energy to acoustic energy. These were simulated in the first step: the frequency domain study. Later, in the second step, the stationary study, the acoustic energy was transformed to fluidic kinetic energy to induce the Eckart jet according to acoustic streaming effects. An inner boundary situation was set to simulate the regulation of a capillary on the jet. The structure to generate BAWs was built according to an actual SMR, and due to fabrication errors, the resonance frequencies of them were a little different. The chamber was set to 3×3 mm^2^, and the outlet of the tube was set to 900 μm wide and 6 mm from the SMR. The other geometrical parameters were mentioned in the specific models. The materials in the 2D models were all consistent with the actual experiments, and from the material library in COMSOL. The pressures of the 3 open boundaries were all set to 0 Pa as initial conditions. The dynamic pressure was calculated at the inlet of the inner boundary situation, which was the closest part to the SMR. The flow velocity was obtained at the outlet of the tube, which was the farthest part to the SMR.

### Characterization of the flow field in an AcousJMP

A glass sink filled with UP water and seeding microspheres with a diameter of 9 to 13 μm (110P8, Lavision GmbH, Germany) was utilized to demonstrate the micropump performance. To fully characterize the steady-state behavior of the micropump under little flow resistances and without external pressure, a wired AcousJMP was immersed in the glass sink and put at a fixed position where its side walls were parallel with the sink wall for observation. Then, the chamber was filled with UP water without any bubbles. Later an RF signal was applied to activate the micropump. A high-speed camera (FASTCAM SA-X2 type 200K-C4, Photron Inc., Japan) was employed to record the movement of particles in the chamber (20,000 frames per second [fps]) and at the outlet of the capillary (125 to 20,000 fps). The flow fields at the different powers in the different inner boundary situations could be obtained in time-lapse photographs from the recorded videos. With the same method, velocities of 5 microspheres in the same cross-section at the outlet of the capillary were obtained, where the fluidic velocity was assumed as a parabolic distribution. The flow rate could be calculated by parabolic fitting of the 5 data points (Fig. 3F).

### Characterization of the pressure delivered in different inner boundary situations

To fully characterize the influence of the inner boundary situation to the pressure, an SMR was placed on a large evaluation board (EVB) (51×15 mm^2^). The EVB was fixed on a chamber with 4 clear side walls for observation and an open top for capillary insertion. Later, the device was immobilized on a 2D displacement platform and connected with a frequency resource (Chengdu Qualwave, China) through an RF line (Fig. S6). Next, the chamber was filled with UP water. A capillary was connected to a silicon tube (external diameter: 2 mm, inner diameter: 1mm), which was fixed in a pipette with a scale. Then the pipette was held by a 1D displacement platform. Utilizing 2 orthometric cameras (B011, Shenzhen Supereyes Technology Co. Ltd, China) (connected to a computer), the SMR was moved directly below the capillary by tuning the 2D displacement platform. Tuning the 1D displacement platform, the distance (*D_i_*) between the SMR and the inlet of the capillary could be changed. A camera was put on the camera holder to record the water level change when power is on. Through analyzing the movie, the water level change could be obtained, and finally the pressure could be estimated as shown in Supplementary Text and Fig. S7.

### Characterization of the pressure delivered by an AcousJMP

To characterize the pressure of an AcousJMP, 2 columnar containers with different diameters were utilized here. The thin and clear container was connected to the outlet of a wired AcousJMP by using a silicon tube (with an outer diameter of 2 mm and an inner diameter of 1 mm). The joints were sealed by UV glue. The container (with a very large inner diameter) was filled with UP water. Then, the AcousJMP was fixed on the bottom of the large container and connected with a signal wire. The AcousJMP was fully filled with UP water and connected to the silicon tube. Then, the UP water entered the tube and reached the same water level as the larger container. When an RF signal was applied, the AcousJMP pumped the water against the gravitational static pressure and the dynamic flow resistance. When the water level gradually became stable, the net flow was nearly zero and the pressure was close to the static pressure. The whole process was recorded by a camera. The height difference between fluidic levels in the thin container and the larger container was obtained through analyzing the movie. Finally, the pressure could be estimated as shown in Supplementary Text and Fig. S7.

### Characterization of droplets generated by an AcousJMP

To characterize the regulation of the geometry of a catheter outlet to the droplets generated by an AcousJMP and the stability of these droplets under different powers, the outlet of the catheter was placed at 2 postures, horizontal and vertical. After connection with an RF signal line, the wired AcousJMP was immobilized using an iron clamp. A silicon tube (with outer diameter of 2 mm) was connected to the inlet of the AcousJMP, and another port was immersed in UP water (Fig. 4G). The outlet of the AcousJMP was jointed with a catheter (with an outer diameter of 1 or 0.8 mm) through a silicon tube (with an inner diameter of 1 or 0.8 mm). Once the AcousJMP was fully filled with water, it was ready to be turned on. A high-speed camera was posited to focus on the outlet, recording the continuous generation of 5 droplets under different powers (2,000 fps). Assuming the droplets were rotational symmetric at the last moment before dropping, the volume of each droplet could be estimated according to its outlines from the frame image (Fig. S10A). Through further analyzing the generation time of the droplet, the flow rate could be obtained (Fig. S10C).

### Animal model of corneal injury and treatment

Adult female SD rats were used in these experiments. The protocol was approved by the Institutional Animal Care and Use Committee of the Tianjin Medical University. The research was conducted in full compliance with the Association for Research in Vision and Ophthalmology statement for the Use of Animals in Ophthalmic and Vision Research. Rats were anesthetized by intraperitoneal injection with a mixture of ketamine (50 mg/kg) and xylazine (10 mg/kg). Next, 100% ethanol was dropped onto the whole cornea for 15 s, which was rinsed with 1 ml 1×PBS later [82,83]. Then, each epithelium over the whole corneal region was mechanically scraped using a surgical blade. The corneal wound was conducted in the bilateral eyes of each rat. The 24 rats were randomly and averagely divided into 3 groups following the performance of injury. Then, the left eyes of the rats in the rhEGF and rhEGF+AcousJMP groups all immediately received topical application of rhEGF eye drops (40,000 IU/4 ml/branch, Guilin Huanowei Genome Pharmaceutical Co. Ltd, China) (by hand and by AcousJMP separately) after the procedure. The eyes of the rats in the PBS group received topical application of 1×PBS. The treatment was topically applied on each eye 4 times per day for a week. The epithelial defect was stained with a topical fluorescent strip and recorded by a slit-lamp microscope under a cobalt blue light (HSL, Weiqing Corp, Suzhou, China) at 0, 12, 24, 48, 72, and 96 h. The injured area of the cornea was determined using the software ImageJ and calculated as a percentage of the residual epithelial defect.

### Histological staining

At the different times after the injury (0, 12, 24, 48, 72, and 96 h), all eyes were enucleated from rhEGF/1×PBS-treated post-injury rats, and the uninjured eyeballs (the control group) were also harvested together. For histological analysis of differentiated corneal and conjunctival tissues, a culture scaffold was gently washed by flushing the perfusion chamber and the open well with 1×PBS. The eyeball was fixed in 4% paraformaldehyde (PFA) solution for 24 h at room temperature. Following with fixations of the eyeball and the scaffold by 4% PFA in 1×PBS overnight at 4 °C, the scaffold was then rinsed with 1×PBS, removed from the device using a scalpel, and transferred into a 6-well plate for further processing. For tissue dehydration, the eyeball was horizontally submerged in 30%, 50%, 70%, 80%, 90%, 95%, and 100% ethanol in sequence. After dehydration, the eyeball was incubated with a clearing agent (CitriSolv, Fisher Scientific, USA) for 30 min and transferred to a 1:1 mixture solution of clearing agent and molten paraffin wax for incubation at 65 °C for 30 min, followed by incubation in 100% molten wax for 60 min. After embedment, the eyeball was sectioned into 5-μm slices and attached to a slide. For H&E staining, a paraffin-embedded section was firstly deparaffinized in the clearing agent and gradually rehydrated in 100%, 95%, 70%, and 50% ethanol. After rinsing in UP water for 1 min, the section was stained with hematoxylin (Sigma-Aldrich, USA) for 1 min and then washed with UP water for 3 min. Then, the section was treated with a bluing reagent (Scott’s buffer) for 20 s, rinsed in UP water for 3 min, and stained with eosin (eosin Y alcoholic, Sigma-Aldrich, USA) for 20 s. Later, the slide was rinsed with 95% ethanol for 1 min twice, 100% ethanol for 1 min, and the clearing agent for 1 min. The final slide was mounted and sealed with a coverslip. Tissue sections stained with H&E were used for observing the corneal epithelium structure and degree of corneal re-epithelization. The sections of the corneal epithelium were measured using ImageJ.

### Participants

The clinical study of volunteers was conducted in Tianjin, China from 2020 October 290 to 2021 February 8. This study was registered on ClinicalTrials.gov (NCT04667819) and followed the Strengthening the Reporting of Observational Studies in Epidemiology guidelines. It is in accordance with the Declaration of Helsinki and is approved by the Ethics Committee of Tianjin Eye Hospital (202076). This study included 12 adults (age: 25 to 36 years) who underwent assessments in Tianjin, China, in 2020. Six patients with DED (12 eyes) wore glasses with AcousJMPs to promote invasive eye drop delivery, while 6 adults (12 eyes) as the control group received the standard-of-care eye drop treatment by hand. The corneal epithelium changes were observed under the slit-lamp microscopy.

### Eye examination

All participants underwent detailed visual examinations including slit-lamp examinations, fundus examinations, and measurements of intraocular pressure using noncontact tonometry (CT-80, Topcon Yamagata Co. Ltd, Japan). The corneal reflection test (Hirschberg test), the Schirmer test, and the TBUT test were also performed. Participants with abnormal findings or strabismus were excluded. The degree of DED was measured using the Schirmer test and the TBUT test. In a Schirmer test, a Schirmer strip was placed in the front of a lower eyelid, and participants were asked to close their eyes for absorption of tears into the Schirmer strip (Tianjin Yinuoxinkang Medical Device Tech Co. Ltd, China). Tear absorption was visualized by the smearing of the blue ink within the strip. Fluorescein stain was conducted in evaluating the corneal epithelium injury in DED patients. After a sterile fluorescein sodium ophthalmic strip (Tianjin Jingming Medical Device Tech Co. Ltd, China) was laid at the base of a scaffold, the eyelid was actuated to blink a few times, during which fluorescein was released from the strip and taken up by contrived tear spreading on the ocular surface. Afterward, the engineered tissue was illuminated under a cobalt blue light and photographed for further analyses. In a TBUT test, a clinical corneal topography instrument (Keratograph 5M, OCULUS, Germany) that could generate illuminated patterns of concentric rings on the ocular surface and monitor their stability for detection of the TBUT was utilized. A single blink in this instrument produced ring patterns on the surface of the engineered tissue that resembled those observed on an ocular surface (Fig. 6C). Quantified data in the graphs are shown as the mean ± standard deviation of TBUTs from 3 independent experiments. *P* values were determined by the 2-sided *t* test. The representative images of eye models are from 3 independent experiments. All tests (TBUT, corneal fluorescein staining, and keratography) were repeated 5 times by experienced ophthalmologists.

### Statistical analysis

The statistical analysis was conducted for measured graphs in each group. To assess whether the TBUT was significantly improved by eye drops, the pre–post comparison was performed using the 2-sided *t* tests. Significant associations were identified using the 2-sided *t* test by comparing results of the normal group and the HL+AcousJMP group. The occurrence of complications for eyes from the same patient was assumed to be independent. The *P* values were obtained from source data and reported in figure legends. The mean difference and its 95% confidence interval were adopted. “ns” means no significant difference (which is omitted in figures), **P < 0.01, ****P < 0.0001.

## Data Availability

All data needed to evaluate the conclusions in the paper are present in the paper and/or the Supplementary Materials.
