# Supplementary material for: A Miniaturized Wireless Micropump Enabled by Confined Acoustic Streaming
Source: Research (Wash D C). 2024 Feb 26;7:0314. doi: 10.34133/research.0314 (PMC10895488; doi:10.34133/research.0314)
Supplement: Supplementary 1 — Supplementary Text Figs. S1 to S12 Tables S1 and S2 Movies S1 to S3 [file research.0314.f1.zip › SUPPLEMENTARY MATERIALS.pdf]

Supplementary Materials for

A Miniaturized Wireless Micropump Enabled by Confined Acoustic Streaming

Rui You[1,†], Qian Fan[2,†], Zilun Wang[1,†], Wenqiang Xing[3,4], Yuchuan Wang[2], Yi Song[2], Yan Wang[2,*], Rui You[3,4,*], Xuexin Duan[1,*]

The PDF file includes:

Supplementary Text
Figures S1 to S12
Tables S1 and S2
Legends for Movies S1 to S3

Other Supplementary Material for this manuscript includes the following:

Movies S1 to S3

Supplementary Text

The calculation of the pressure.

The pressure of pumping is mainly from the dynamic flow resistance and the static gravitational pressure, which can be expressed as [*52*]:

$$p(t) = \rho g h(t) + RQ(t) \tag{6}$$

where *p(t)* is the pressure caused by the actuator, *i.e.*, the pump; ρ is the density of the fluid; g is the gravitational acceleration; *h* is the height difference between fluidic levels, *R* is the flow resistance; *Q* is the flow rate. Because of the Reynolds number, *Re*, is low in the fluidic field, it can be assumed the flow is pressure-driven and laminar, *i.e.*, a Poiseuille flow. Thus, the flow resistance can be calculated as:

$$R = R_0 + R(t) = \frac{128\mu L_1}{\pi D_1^4} + \frac{128\mu L_2}{\pi D_2^4} + \frac{128\mu L(t)}{\pi D_3^4} \tag{7}$$

where R_0 is a constant and from the part immersed in the fluid; *R(t)* is from the part above the low fluidic level, varying with the time; *D* and L are the diameter and the length of the corresponding tube in the pump, respectively. In this paper, D_1 is about 0.1-0.2 mm, D_2 is 0.8 mm, and D_3 is 1 mm. While L_1 is about 1 mm, L_2 is about 2-3 mm, and *L(t)* is less than 400 mm. Thus, we can assume $R(t) << R_0$, so $R \approx R_0$, and *Q(t)* can be expressed through *h(t)*:

$$Q(t) = h'(t)A = h'(t)\frac{\pi D_3^2}{4} \tag{8}$$

where *A* is the cross-sectional area of the tube above the low fluid level. Then the Eq. 6 can be further written as:

$$p(t) = RAh'(t) + \rho g h(t) \tag{9}$$

Then through solving Eq. 9, *h(t)* can be described as:

$$h(t) = \frac{p_0}{\rho g}(1 - e^{-\rho g t/C_0}) \tag{10}$$

where p_0 and C_0 are artificial parameters, related to the physical quantities by $p_0/C_0 = p(t)/C|_{t=0}$. Besides, *C* is an artificial parameter to tune *R*, rewriting $R(t) = C/A$ to produce a least-squares fits of *h(t), p(t)* to the experimental data. Substitution of *h(t)* and *R(t)* in Eq. 9, *p(t)* can be rewritten as:

$$p(t) = p_0 + p_0 e^{-\rho g t/C_0}(\frac{C}{C_0} - 1) \tag{11}$$

Through further analyzing Eq. 10 and Eq.11, the pressure can be estimated as:

$$p(\infty) = \rho g h(\infty) = p_s(\infty) \tag{12}$$

In another word, the pressure will be equal to the static pressure when the system reaches a steady state. For example, in the situation of a conical-port capillary with a 140-µm bottom width, and 45-µm bottom height, the experimental points and the fitting curves of static pressure at 50, 100, 500 and 1000 mW, $p_s(t)$, were plotted as Fig. S7, and each R^2 is larger than 0.99.

Figure S1. Fabrication processes of a solid-mounted film bulk acoustic wave resonator (SMR).

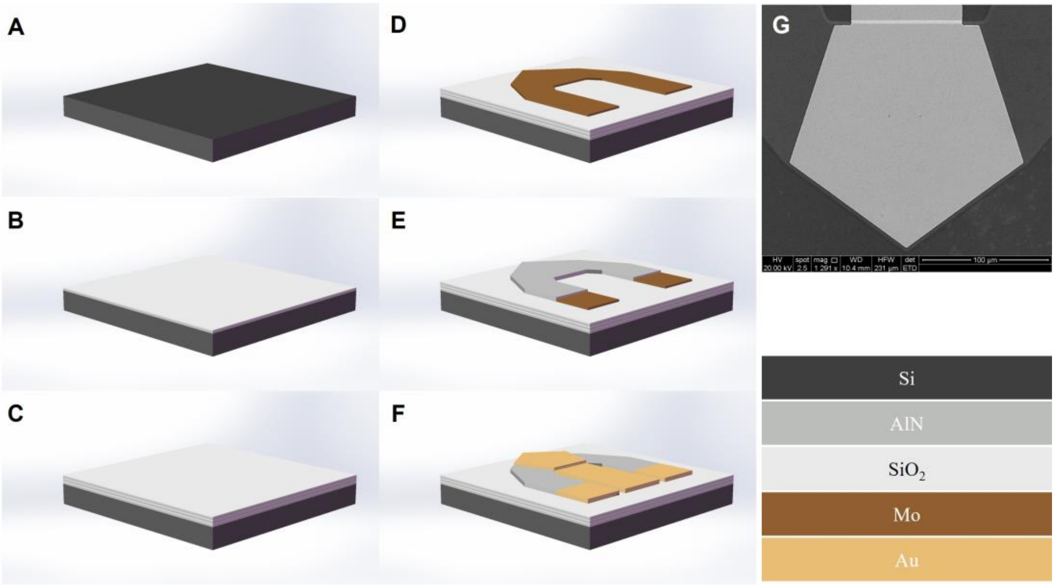

Fig. S1. Fabrication processes of an SMR. (A) 4-inch silicon wafer cleaning by piranha solution; (B-C) Bragg reflector layer mounted on the wafer by alternatively depositing AlN and SiO_2 (1.2 um, 700 nm, 1 um, 1.3 um, 1 um, 650 nm in order); (D) Bottom electrode (Mo, 600 nm) patterned on the Bragg reflector; (E) Piezoelectric layer (AlN, 1 µm) patterned on the bottom electrode; (F) Top electrode patterned (Au, 300 nm) on the piezoelectric layer, and two electrodes patterned on the bottom electrode for later connection. (G) SEM photograph of an SMR with a resonance area of ~20 kμm^2.

Figure S2. Simulation results.

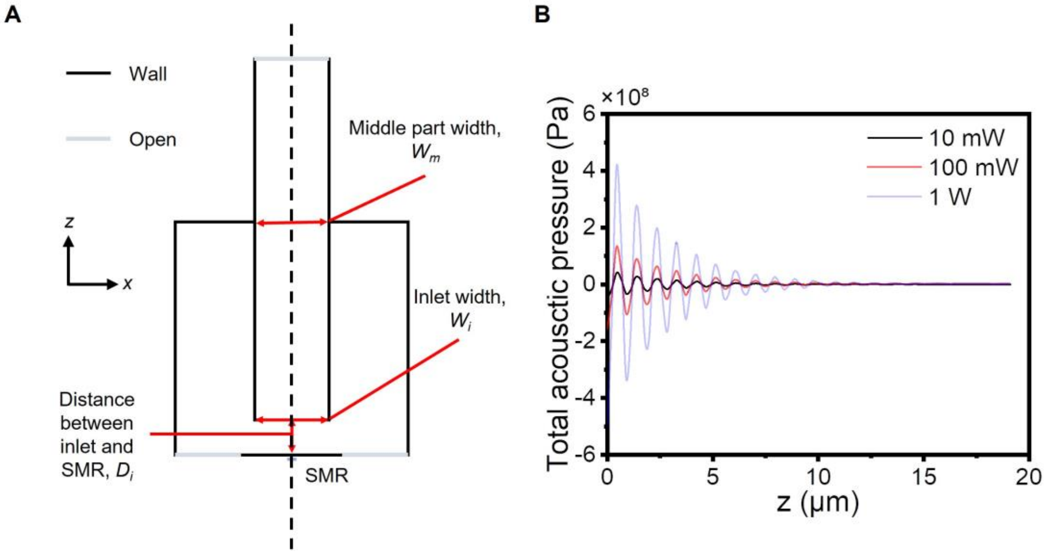

Fig. S2. Simulation results of the attenuation of 1.596 GHz acoustic waves in water. (A) Geometry of a 2D FEM model consists of a square chamber of 3×3 mm^2, a SMR in the middle under the chamber and a capillary with an outlet of 0.9 mm wide and 6 mm from the SMR. The chamber has two 0.9 mm wide open boundaries at two sides of the bottom. The capillary has two symmetric inner boundaries in the overlap with the chamber. (B) Attenuation of 1.596 GHz bulk acoustic waves in the water along the *z*-axis.

Figure S3. Simulation flow fields of slit and conical capillaries.

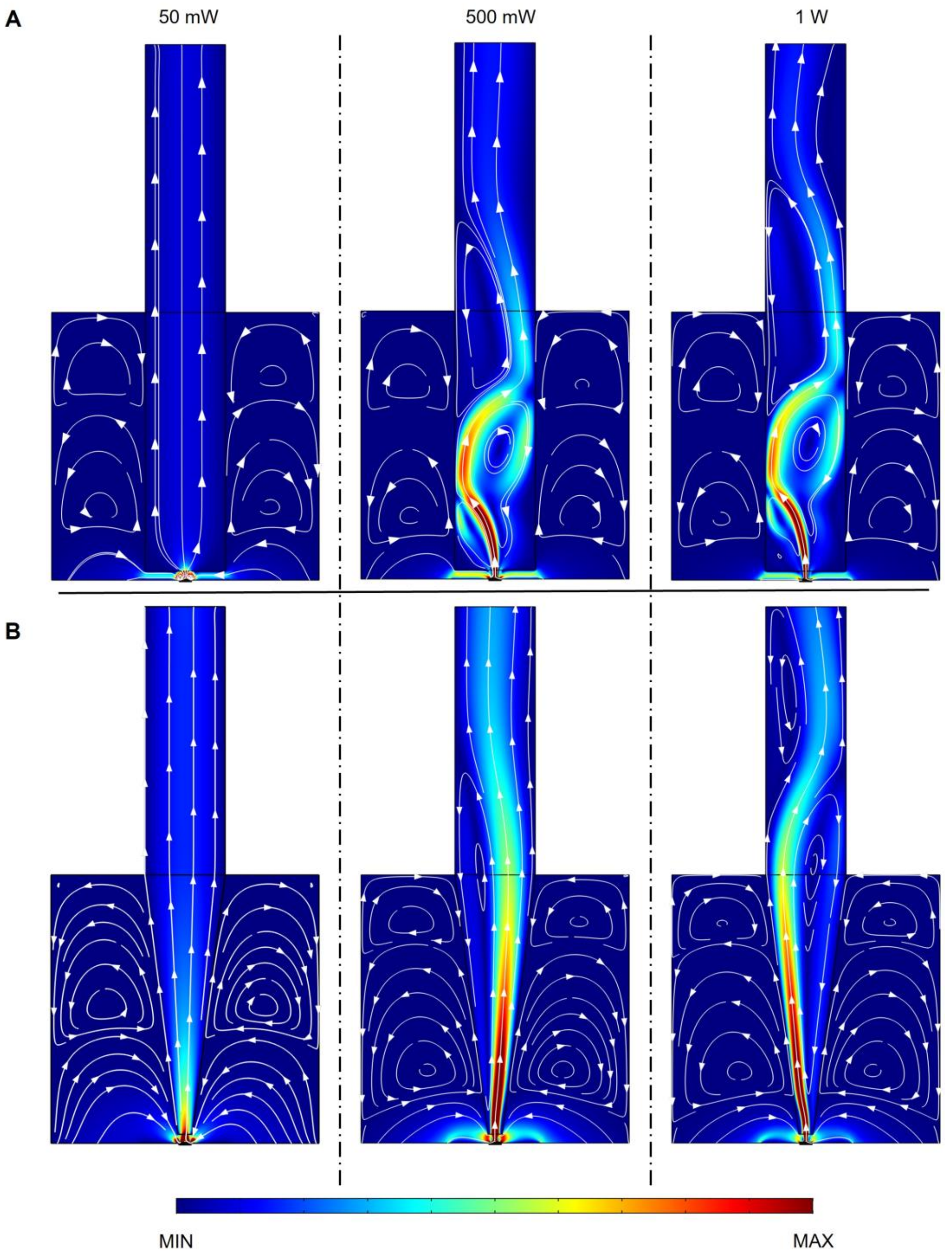

Fig. S3. Regulation of inner boundary situations to the flow field. (A) Slit capillary with an open inlet of 160 μm. (B) Conical capillary with an inlet of 160 μm wide and a distance of 100 μm from the SMR.

Figure S4. Simulation flow fields of straight and conical capillaries.

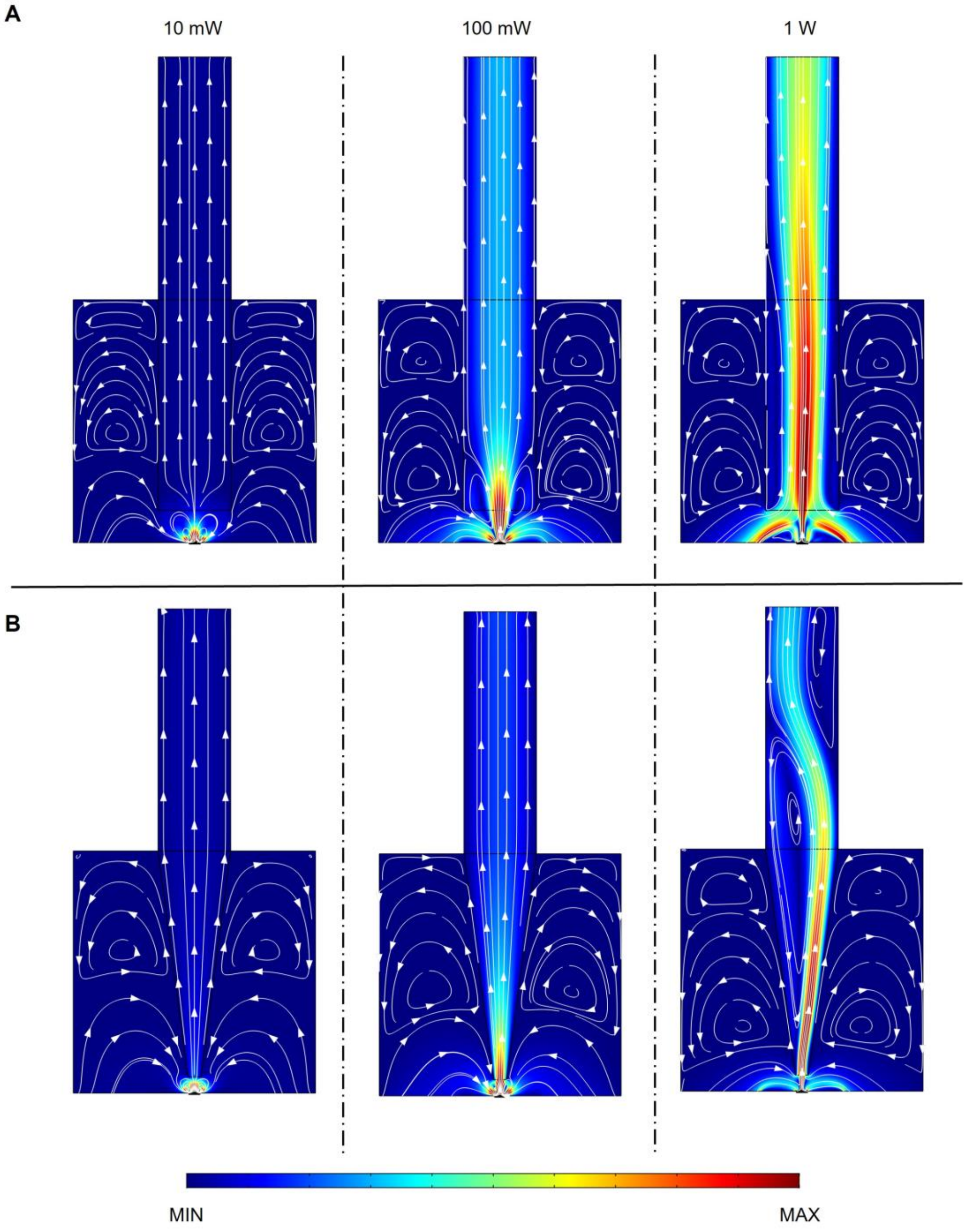

Fig. S4. Regulation of inner boundary situations to the flow field. (A) Straight capillary with a distance of 160 μm from the SMR. (B) Conical capillary with an inlet of 160 μm and a distance of 200 μm from the SMR.

Figure S5. Pumping performances of AcousJMPs with different boundary situations.

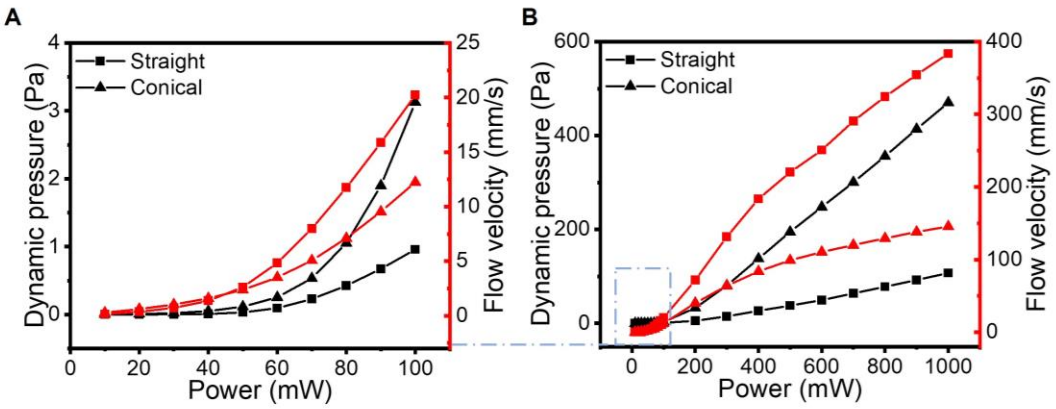

Fig. S5. Changes of dynamic pressure and flow rate in different inner boundary situations when the power varies.

Figure S6. Setup of the static pressure test for different inner boundary situations.

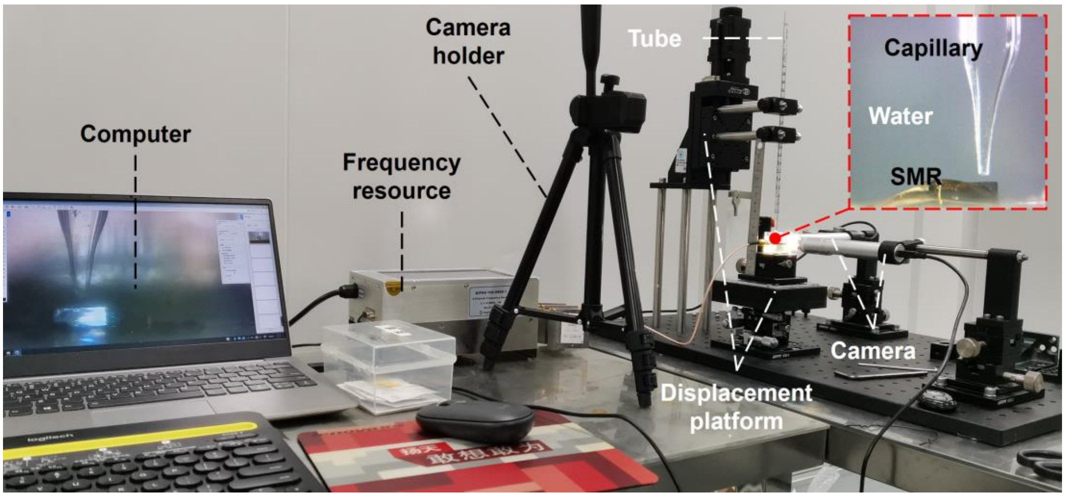

Fig. S6. Photograph of the detailed experimental setup for the static pressure test for different inner boundary situations, including two displacement platforms for the alignment of a capillary and an SMR, two orthometric cameras and a computer for observation and assistance with the alignment, a frequency resource for power supply, a pipette with scale for measurement, and a camera and a camera holder for recording.

Figure S7. Fitting of static pressure.

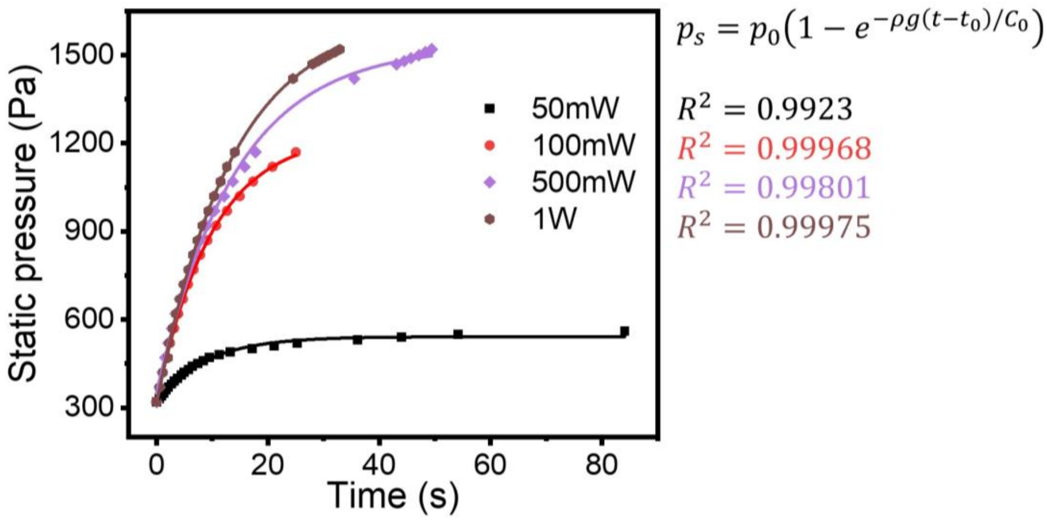

Fig. S7. Fitting of static pressure for the estimation of pressure.

Figure. S8. Static pressure tests for AcousJMPs with different capillaries.

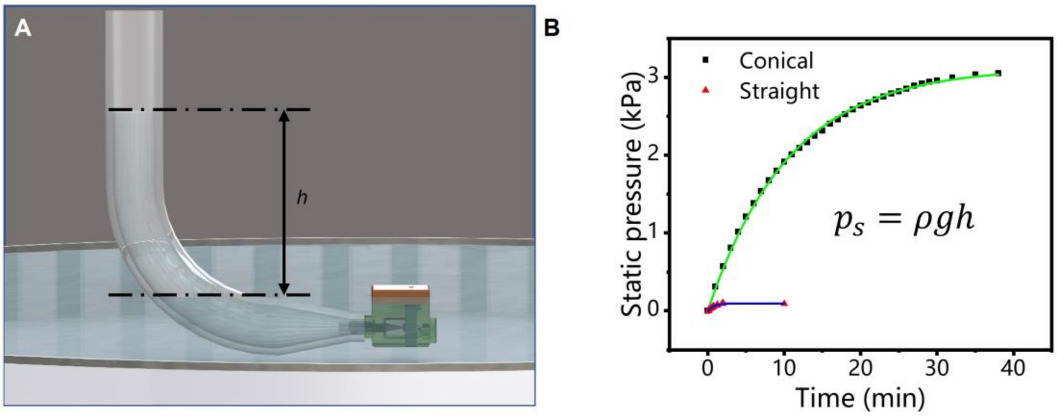

Fig. S8. Static pressure tests for AcousJMPs. (A) Schematic illustration of the static pressure test for the AcousJMP. (B) Static pressure changes of AcousJMPs with straight and conical capillaries respectively.

Figure. S9. Performances of AcousJMP I.

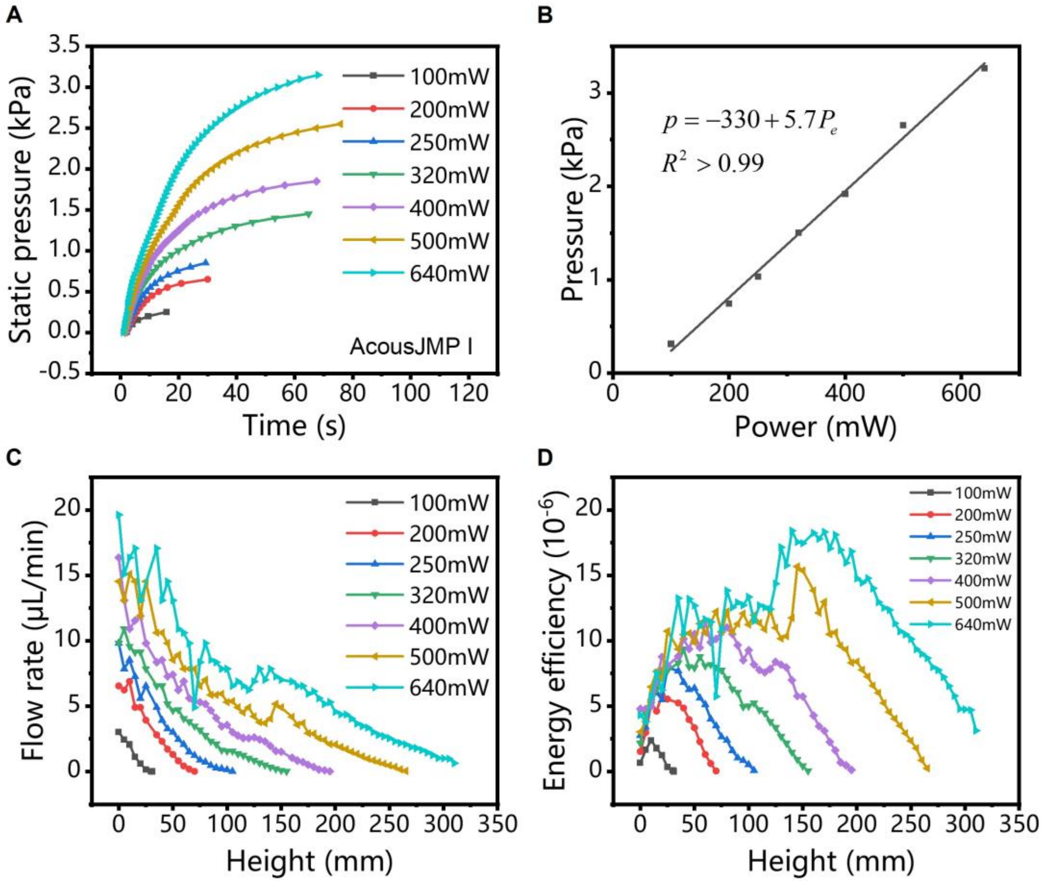

Fig. S9. Pumping performance of AcousJMP I. (A) Static pressure changes of AcousJMP I. (B) Estimated backpressure of AcousJMP I at different powers. (C) Flow rate curves of AcousJMP I as the different heights change under different powers. (D) Energy efficiency curves of AcousJMP I as the different heights change under different powers.

Figure S10. Droplets generated by different outlet geometries.

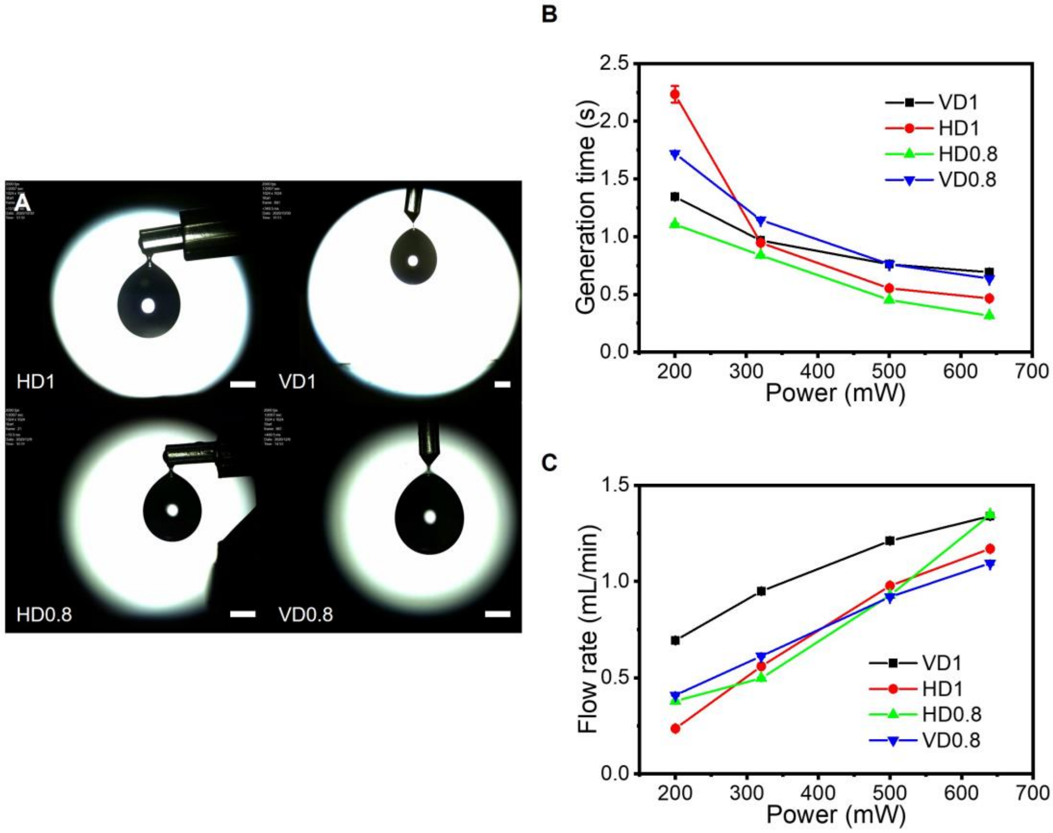

Fig. S10. (A) Droplets recorded by a high-speed camera under different outlet geometries. Scale bar: 1 mm. (B) Droplet generation time changes with the applied power. (C) Flow rate of the AcousJMP with different catheter interfaces.

Figure S11. Thermal test of a wireless AcousJMP.

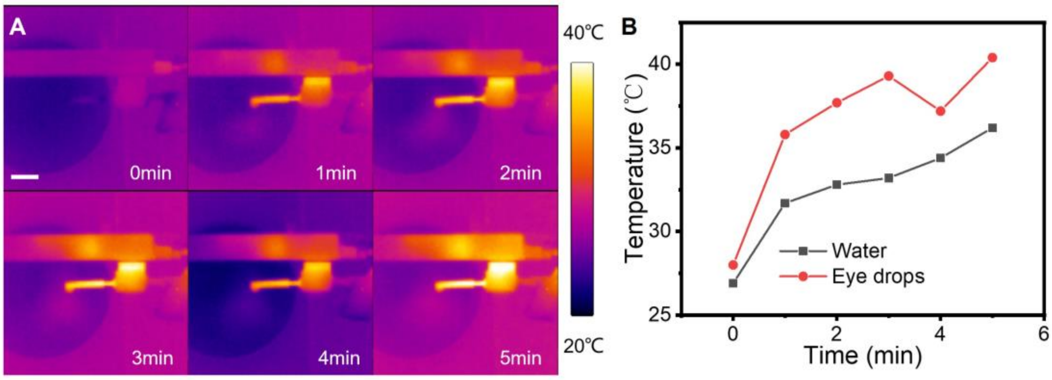

Fig. S11. (A) IR images of a wireless AcousJMP pumping eyedrops out of a catheter. Scale bar: 1 cm. (B) Maximum temperature changes of the working AcousJMP filled with water and eye drops.

Figure S12. Portable external terminal.

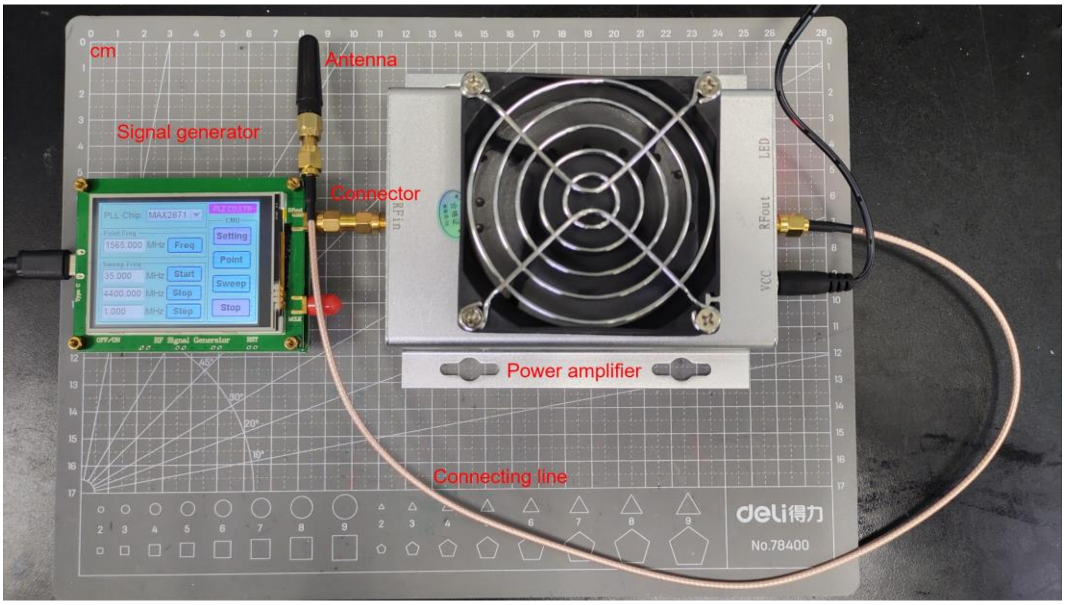

Fig. S12. Photograph of the portable external terminal consisting of a signal generator, a power amplifier, an antenna, a connector, a connecting line and two DC adaptors.

Table S1. Comparison of micropump performance

Reference-year	Pumping principle	Approximate size (cm^3)	Power supple	Max. pressure (kPa)	Max. flow rate (mL/min)	Pressure / size (kPa/cm^3)	Flow rate / size ($mL/min·cm^3$)
(*48*)-2002	electroosmotic	9.000	1 kV	33.430	0.015	3.714	0.002
(*28*)-2005	thenrmo-pneumatic	1.170	20 V	1	0.022	0.855	0.018
(*30*)-2010	piezoelectric	70.000	250 V	N/A	6.400	N/A	0.091
(*42*)-2011	electromagnetic	0.840	0.18 A	0.950	0.320	1.131	0.381
(*46*)-2011	phase change	1.568	N/A	28.900	0.029	18.431	0.018
(*29*)-2012	piezoelectric	1.760	110 V	N/A	0.152	N/A	0.086
(*45*)-2012	electromagnetic	2.400	7.5 V	N/A	0.130	N/A	0.054
(*31*)-2012	piezoelectric	75.000	320 V	0.930	23.000	0.012	0.307
(*32*)-2013	piezoelectric	1.080	100 V	1.550	0.630	1.435	0.583
(*43*)-2016	electromagnetic	0.144	6 V	0.245	0.135	1.701	0.938
(*47*)-2016	dielectric elastomer	8.400	4.2 kV	N/A	2.520	N/A	0.300
(*33*)-2017	piezoelectric	0.144	200 V	2.350	0.240	16.319	1.667
(*38*)-2017	electromagnetic	2.800	N/A	0.750	0.700	0.268	0.250
(*41*)-2017	piezoelectric	405.000	7.5 V	4.390	0.497	0.011	0.001
(*34*)-2018	piezoelectric	33.000	20 V	0.245	30.700	0.007	0.930
(*49*)-2019	electrohydrodynamic	1.170	8 kV	14.000	6.000	11.966	5.128
(*40*)-2019	piezoelectric	3.600	210 V	N/A	3.600	N/A	1.000
(*39*) - 2019	piezoelectric	25.200	220 V	N/A	286.000	N/A	11.349
(*37*) - 2019	piezoelectric	79.380	190 V	0.670	220.600	0.008	2.779

(*36*) - 2019	piezoelectric	154.375	300 V	N/A	8.700	N/A	0.056
(*35*)- 2020	piezoelectric	1.866	150 V	0.294	7.192	0.158	3.855
(*44*)- 2021	electromagnetic	11.700	4.1 W	0.3	30.600	0.026	2.615
AcousJMP I	acoustofluidic	0.100	0.64 W	3.262	1.180	32.620	11.800

Red: parameters of AcousJMP I, and parameters better than those of AcousJMP I.

Table S2. Comparison of acoustofluidic micropump performance

Reference-year	Power supply	Frequency (MHz)	Max. pressure (Pa)	Max. flow rate (μL/min)
(53)-2009	40 V	N/A	170	0.25
(52)-2014	2.21 W	122	16.4	1570
(50)-2014	50 V	6.5×10^{-3}	76	8
(51)-2019	6 W	10.68	N/A	0.0415
(23)-2019	4 V	0.099	N/A	0.42
(54)-2021	40 V	0.06373	N/A	12
(55)-2021	105 V	2.07	37.6	6400
AcousJMP I	0.64 W	1540	3262	1180

Red: parameters of AcousJMP I, and parameters better than those of AcousJMP I.

Supplementary Movies

Movie S1. Assembly of a wireless AcousJMP.

Movie S2. rhEGF delivered by wireless AcousJMP onto rat's ocular surface.

Movie S3. Eye drop delivered by wireless AcousJMP onto patient's ocular surface.
