# Supplementary figures and images for: A Miniaturized Wireless Micropump Enabled by Confined Acoustic Streaming
Source: Research (Wash D C). 2024 Feb 26;7:0314. doi: 10.34133/research.0314 (PMC10895488; doi:10.34133/research.0314)

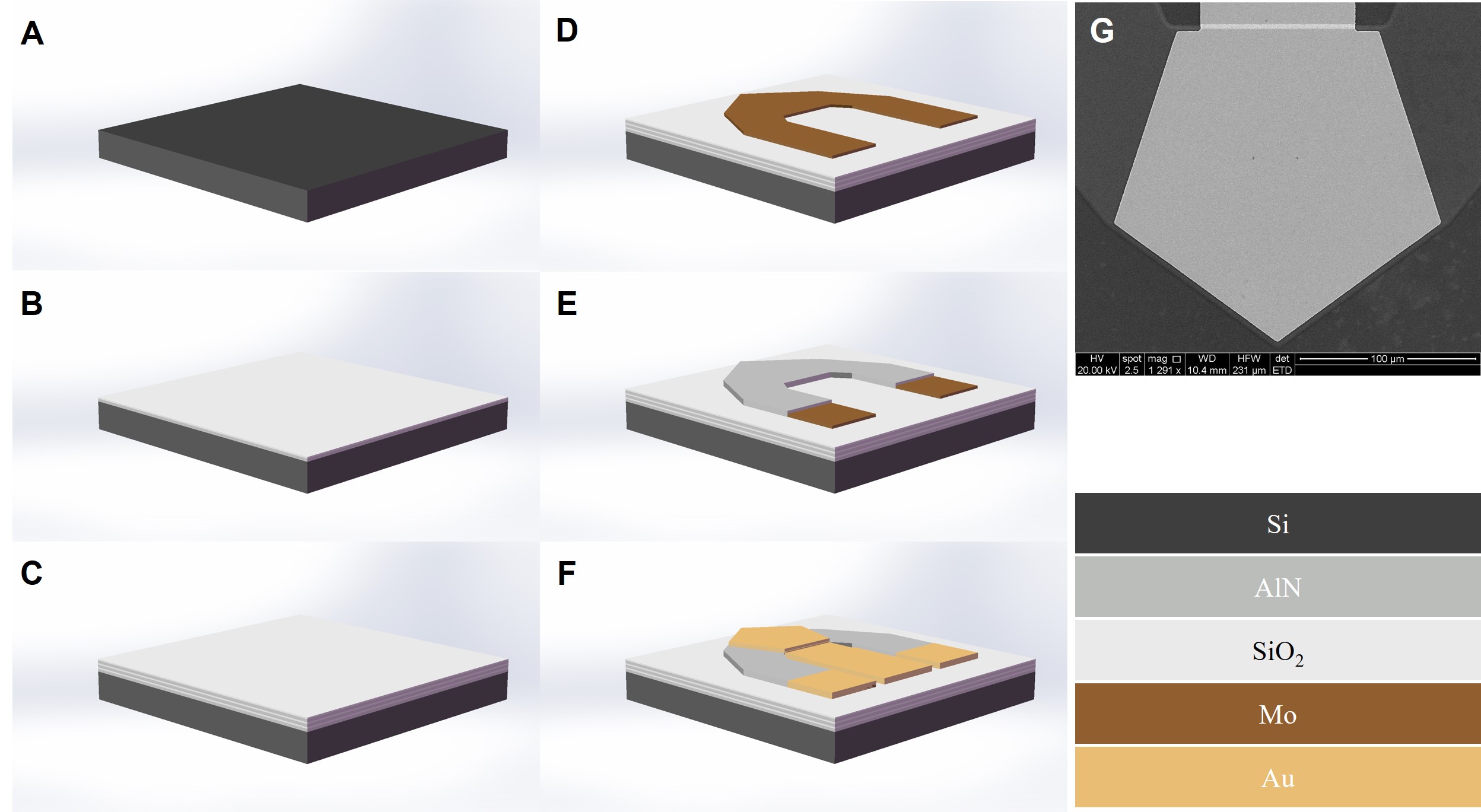

Supplement: Supplementary 1 — Supplementary Text Figs. S1 to S12 Tables S1 and S2 Movies S1 to S3 [file research.0314.f1.zip › s1.jpg]

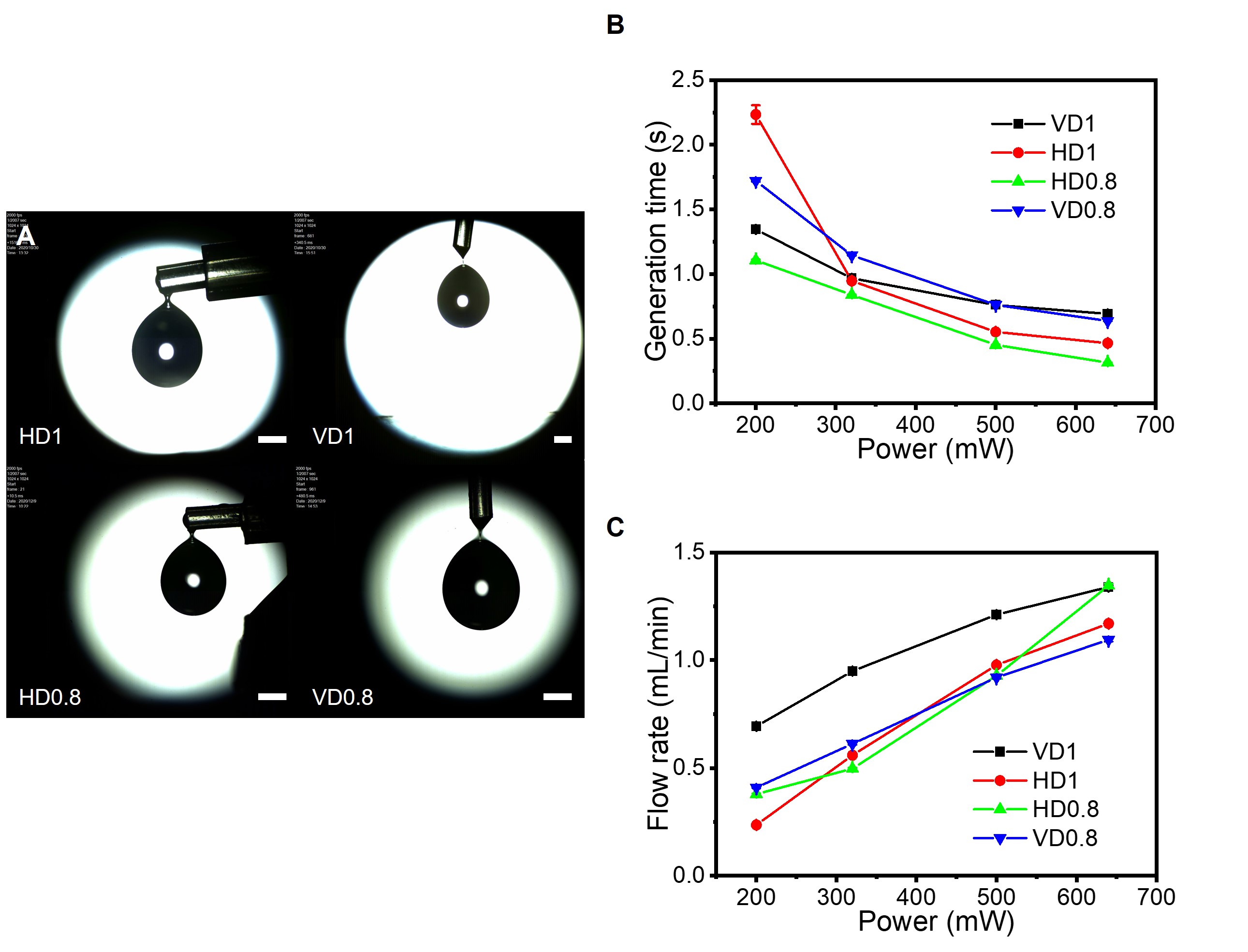

Supplement: Supplementary 1 — Supplementary Text Figs. S1 to S12 Tables S1 and S2 Movies S1 to S3 [file research.0314.f1.zip › s10.jpg]

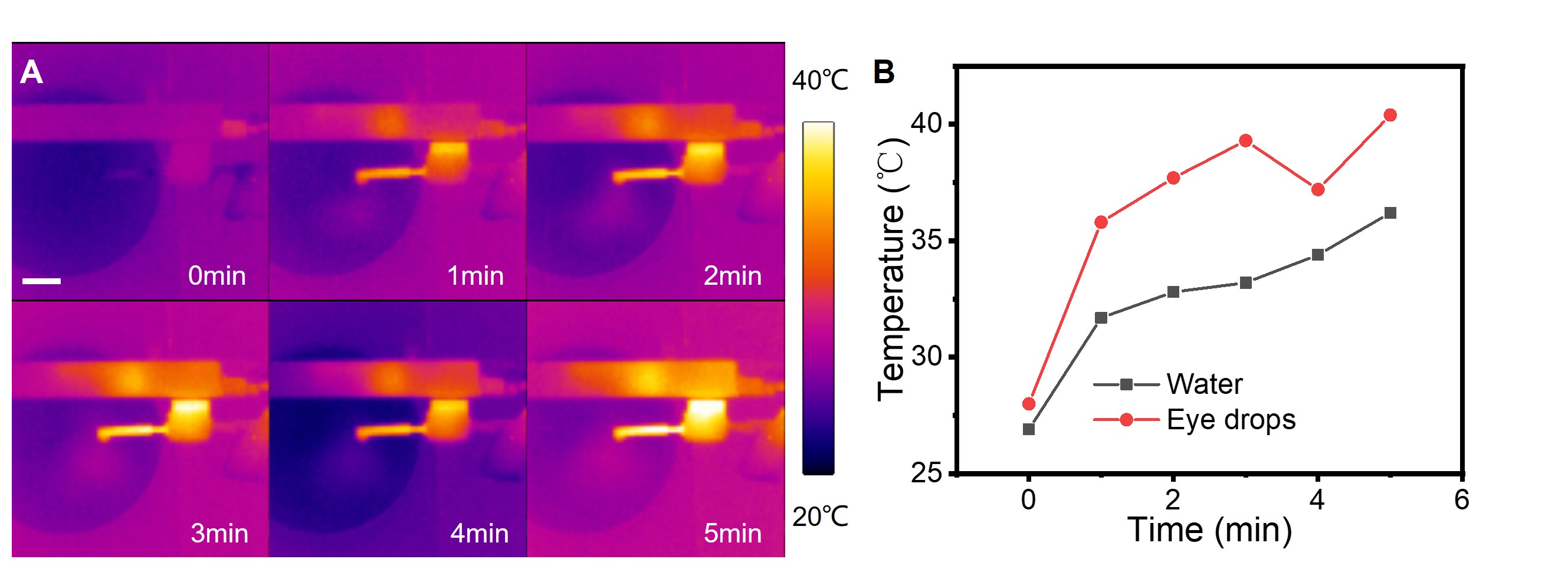

Supplement: Supplementary 1 — Supplementary Text Figs. S1 to S12 Tables S1 and S2 Movies S1 to S3 [file research.0314.f1.zip › s11.jpg]

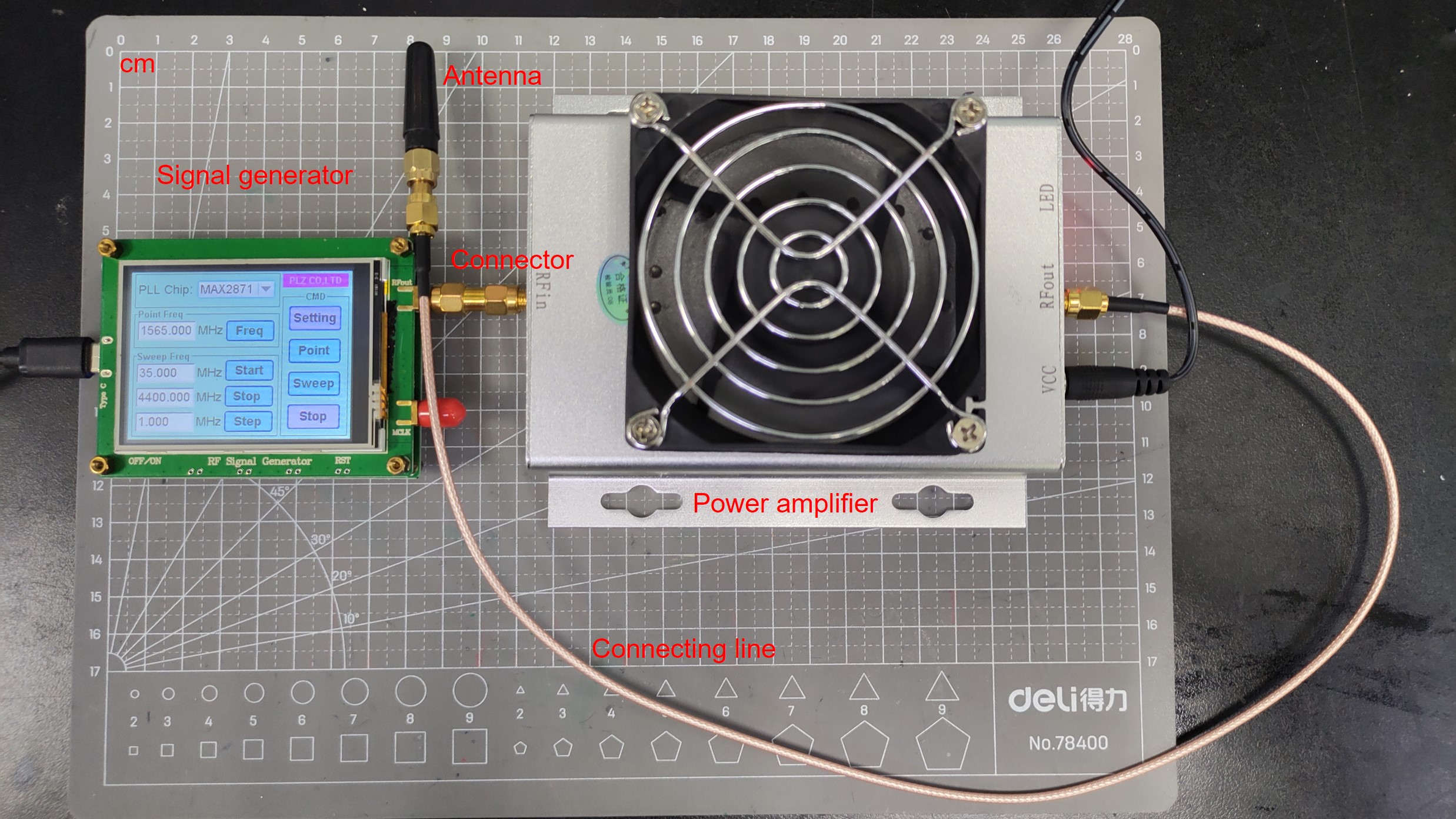

Supplement: Supplementary 1 — Supplementary Text Figs. S1 to S12 Tables S1 and S2 Movies S1 to S3 [file research.0314.f1.zip › s12.jpg]

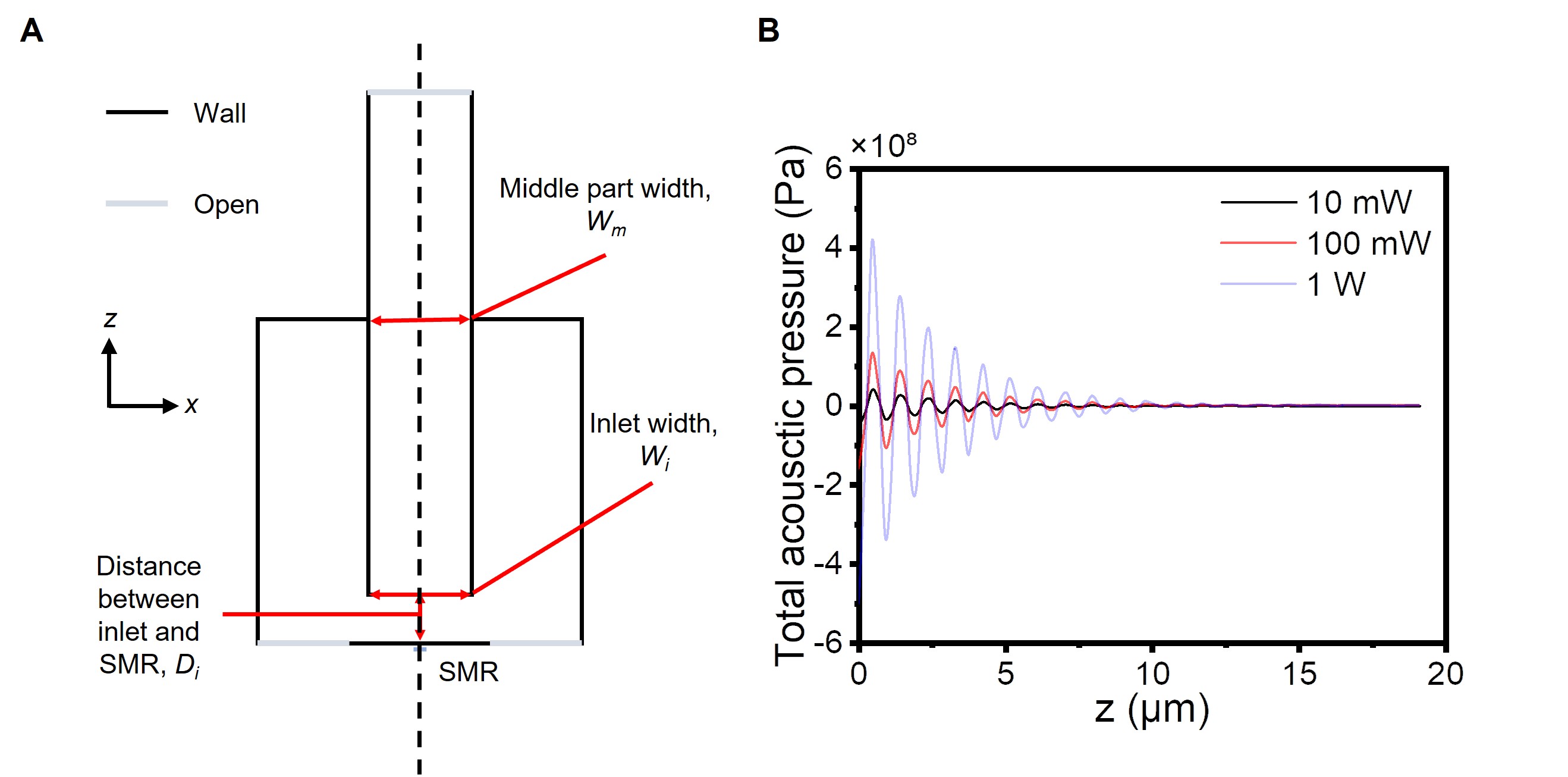

Supplement: Supplementary 1 — Supplementary Text Figs. S1 to S12 Tables S1 and S2 Movies S1 to S3 [file research.0314.f1.zip › s2.jpg]

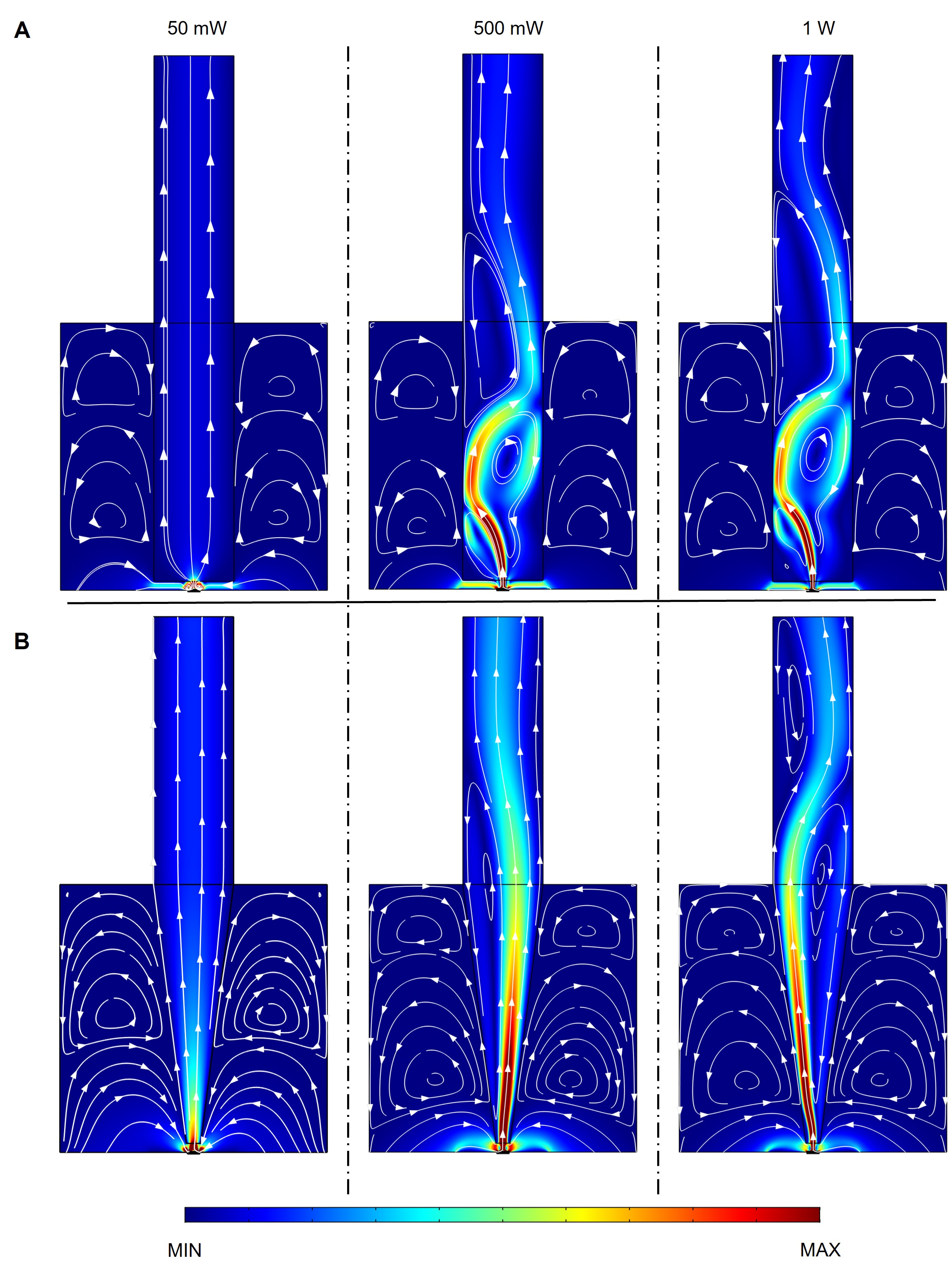

Supplement: Supplementary 1 — Supplementary Text Figs. S1 to S12 Tables S1 and S2 Movies S1 to S3 [file research.0314.f1.zip › s3.jpg]

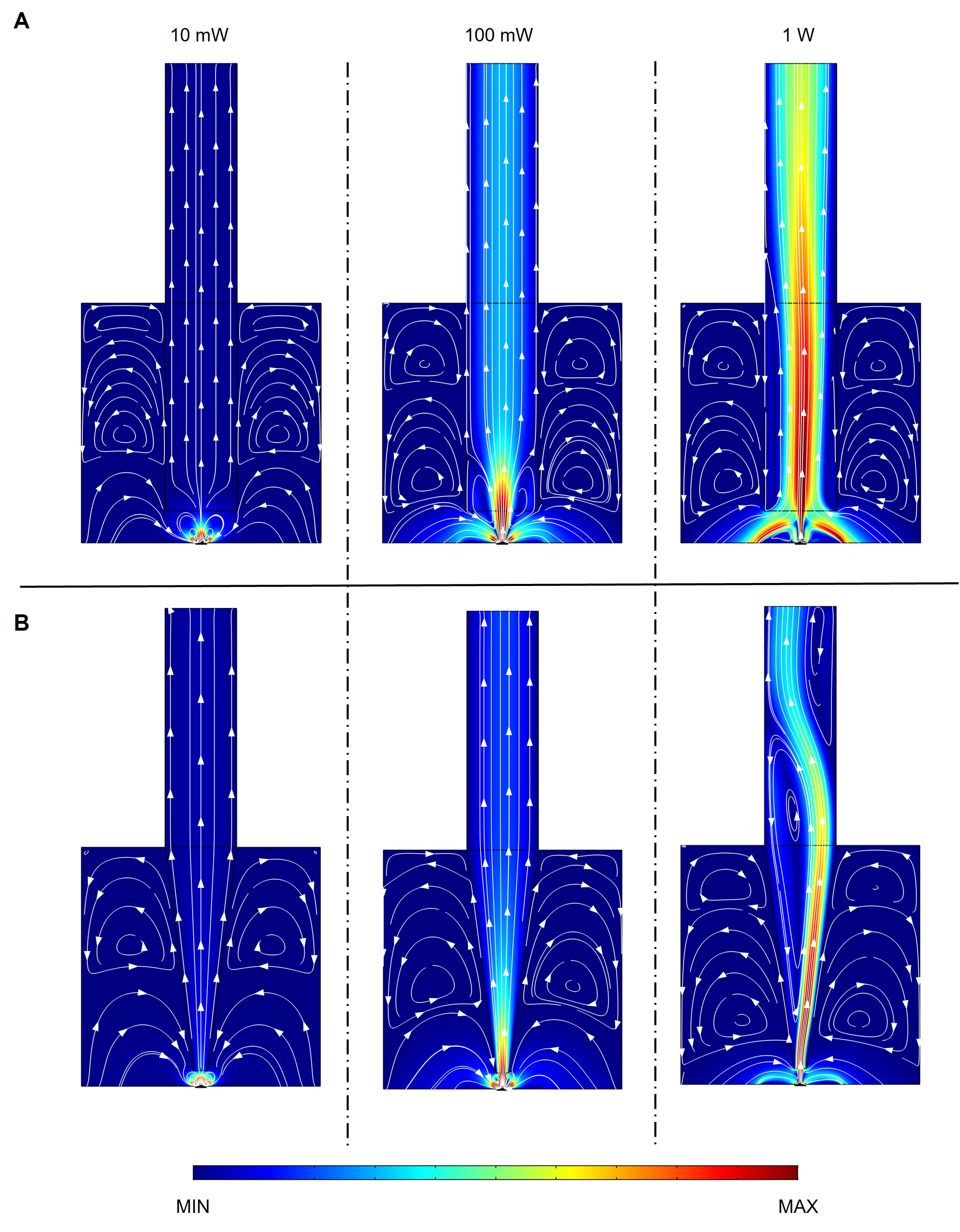

Supplement: Supplementary 1 — Supplementary Text Figs. S1 to S12 Tables S1 and S2 Movies S1 to S3 [file research.0314.f1.zip › s4.jpg]

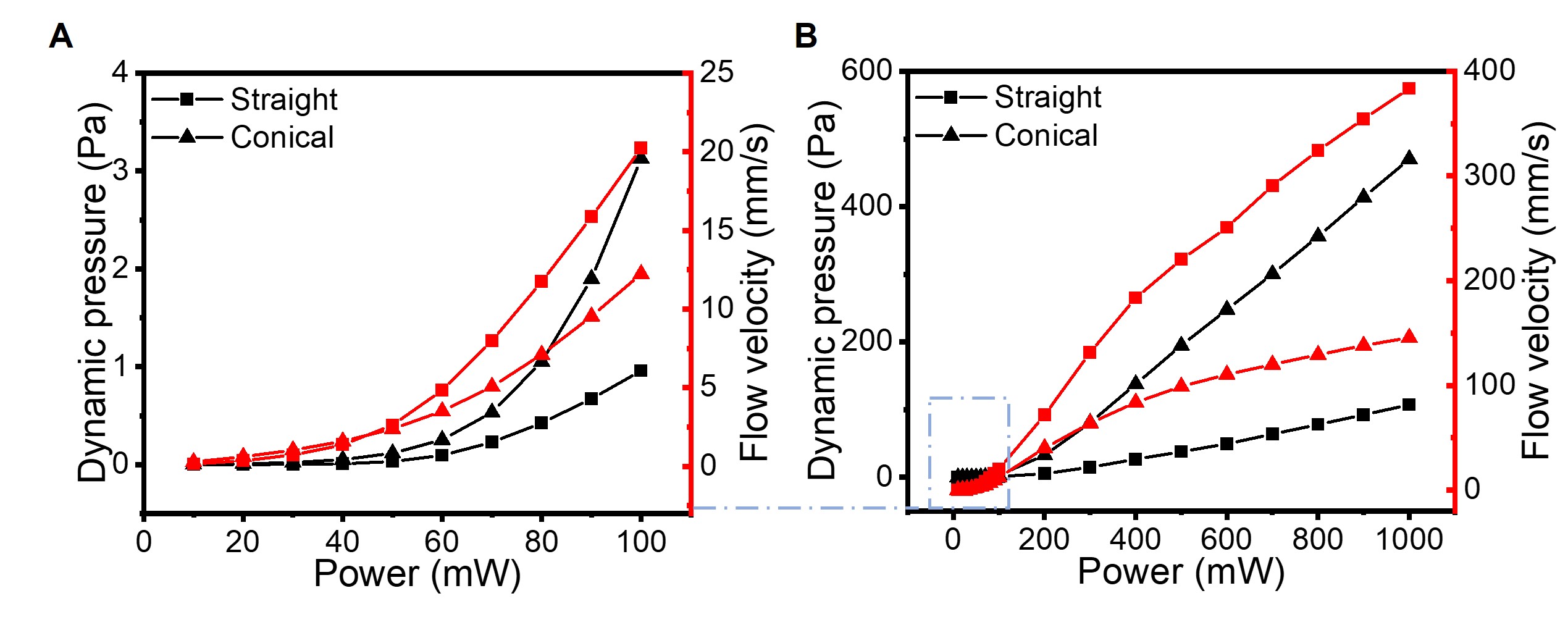

Supplement: Supplementary 1 — Supplementary Text Figs. S1 to S12 Tables S1 and S2 Movies S1 to S3 [file research.0314.f1.zip › s5.jpg]

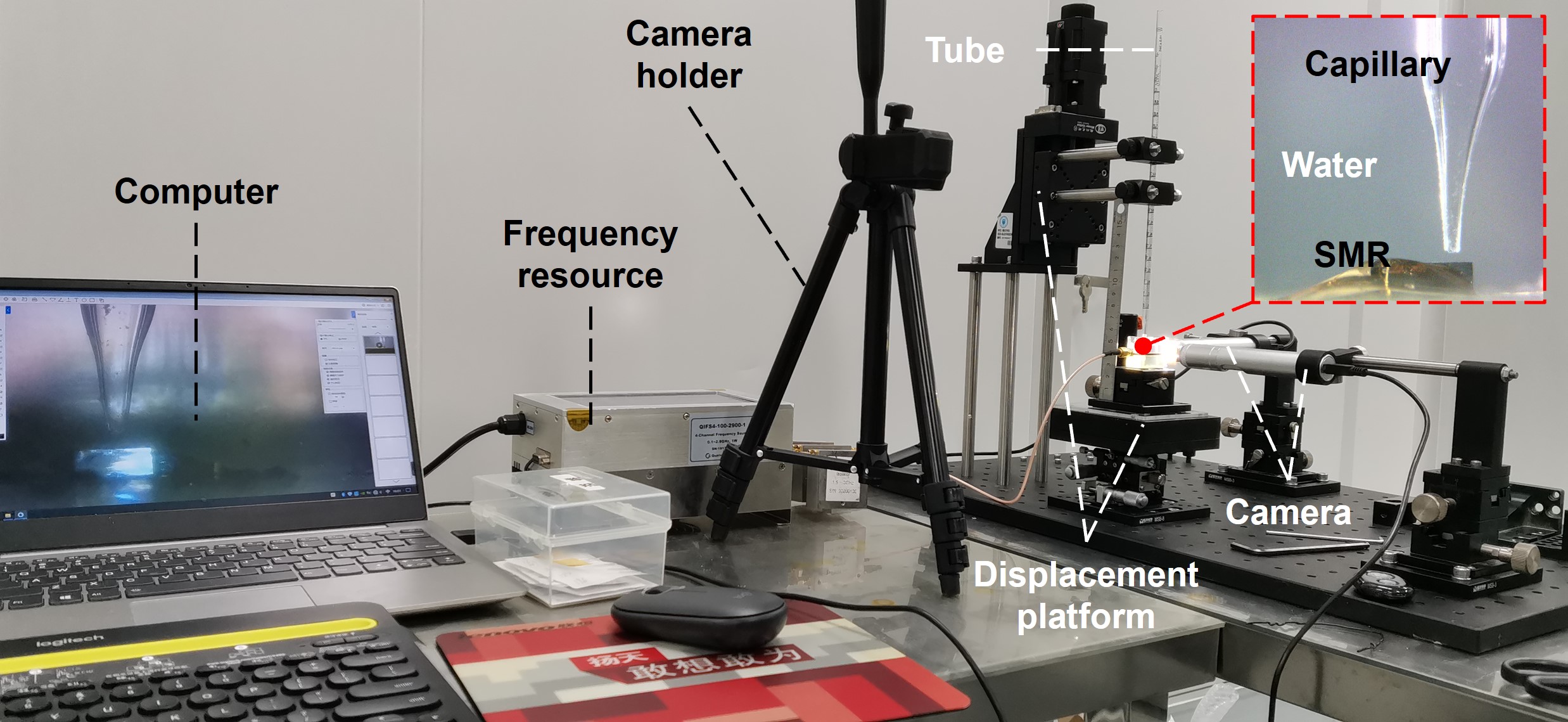

Supplement: Supplementary 1 — Supplementary Text Figs. S1 to S12 Tables S1 and S2 Movies S1 to S3 [file research.0314.f1.zip › s6.jpg]

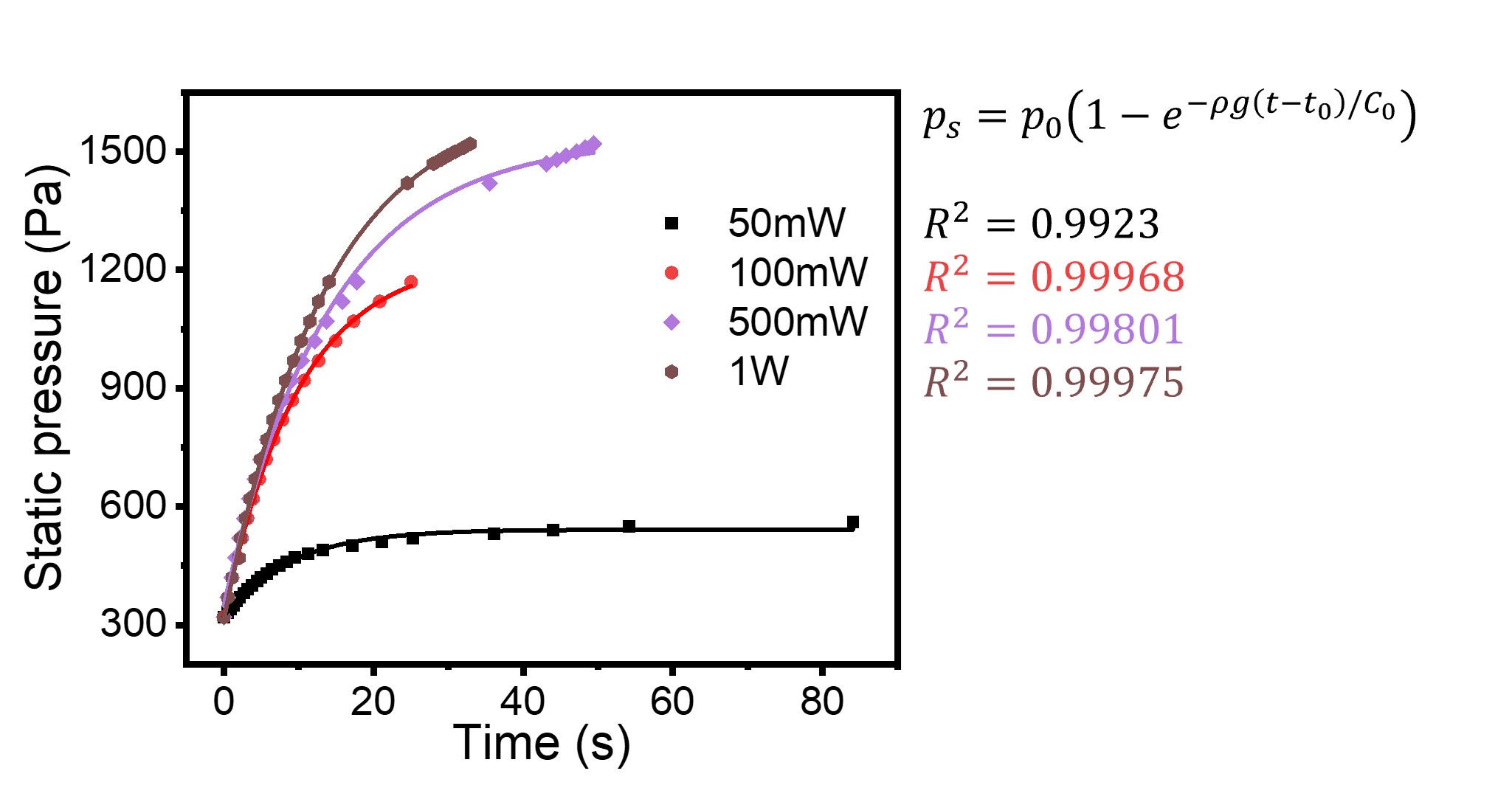

Supplement: Supplementary 1 — Supplementary Text Figs. S1 to S12 Tables S1 and S2 Movies S1 to S3 [file research.0314.f1.zip › s7.jpg]

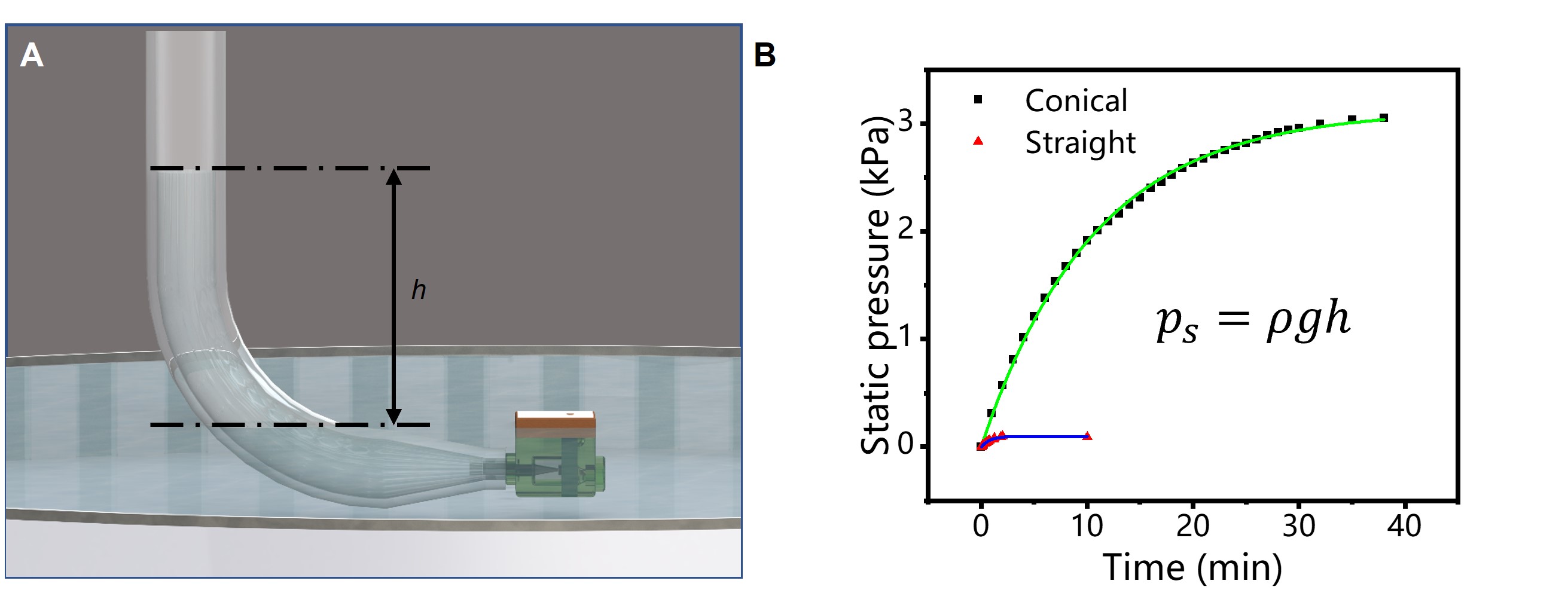

Supplement: Supplementary 1 — Supplementary Text Figs. S1 to S12 Tables S1 and S2 Movies S1 to S3 [file research.0314.f1.zip › s8.jpg]

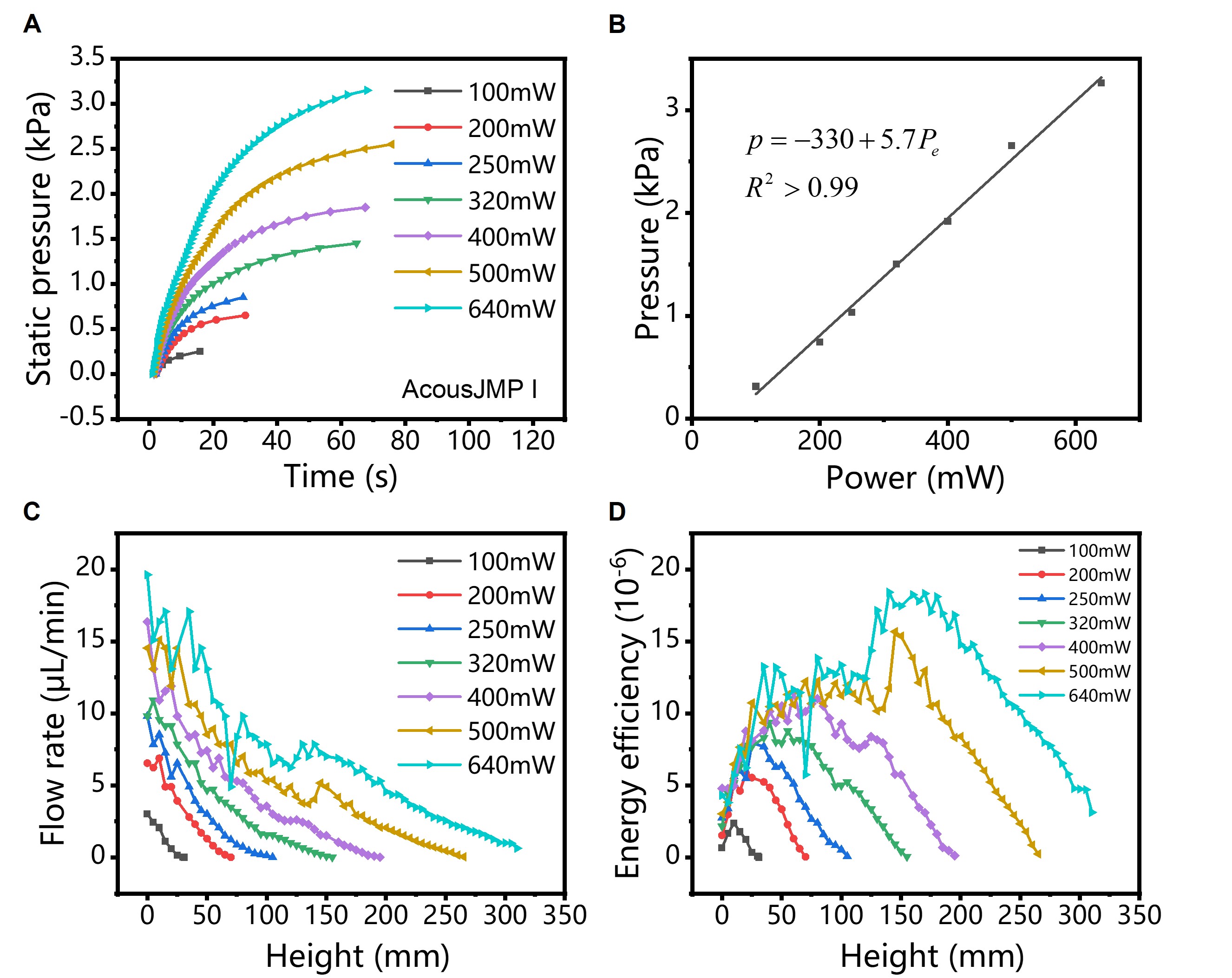

Supplement: Supplementary 1 — Supplementary Text Figs. S1 to S12 Tables S1 and S2 Movies S1 to S3 [file research.0314.f1.zip › S9.jpg]
